# Thoracic and cardiovascular surgery in Japan during 2014: Annual report by The Japanese Association for Thoracic Surgery

**DOI:** 10.1007/s11748-016-0695-3

**Published:** 2016-09-02

**Authors:** Committee for Scientific Affairs, The Japanese Association for Thoracic Surgery, Munetaka Masuda, Meinoshin Okumura, Yuichiro Doki, Shunsuke Endo, Yasutaka Hirata, Junjiro Kobayashi, Hiroyuki Kuwano, Noboru Motomura, Hiroshi Nishida, Yoshikatsu Saiki, Aya Saito, Hideyuki Shimizu, Fumihiro Tanaka, Kazuo Tanemoto, Yasushi Toh, Hiroyuki Tsukihara, Shinji Wakui, Hiroyasu Yokomise

**Affiliations:** 1 Tokyo, Japan; 2 Department of Surgery, Yokohama City University, Yokohama, Japan; 3 Department of General Thoracic Surgery, Osaka University Graduate School of Medicine, Osaka, Japan; 4 Department of Gastroenterological Surgery, Osaka University Graduate School of Medicine, Osaka, Japan; 5 Department of Thoracic Surgery, Jichi Medical University, Tochigi, Japan; 6 Department of Cardiac Surgery, The University of Tokyo Hospital, Tokyo, Japan; 7 Department of Cardiovascular Surgery, National Cerebral and Cardiovascular Center, Osaka, Japan; 8 Department of General Surgical Science Graduate School of Medicine, Gunma University, Gunma, Japan; 9 Department of Cardiovascular Surgery, Sakura Medical Center, Toho University, Chiba, Japan; 10 Department of Cardiovascular Surgery, The Heart Institute of Japan, Tokyo Women’s Medical University, Tokyo, Japan; 11 Division of Cardiovascular Surgery, Tohoku University Graduate School of Medicine, Miyagi, Japan; 12 Department of Cardiovascular Surgery, Keio University, Tokyo, Japan; 13 Second Department of Surgery, University of Occupational and Environmental Health, Fukuoka, Japan; 14 Department of Cardiovascular Surgery, Kawasaki Medical School, Okayama, Japan; 15 Department of Gastroenterological Surgery, National Kyushu Cancer Center, Fukuoka, Japan; 16 Department of Cardiothoracic Surgery, Graduate School of Medicine, The University of Tokyo, Tokyo, Japan; 17 Department of Cardiovascular Surgery, Nihon University Hospital, Tokyo, Japan; 18 Department of General Thoracic Surgery, Faculty of Medicine, Kagawa University, Kagawa, Japan

The Japanese Association for Thoracic Surgery has conducted annual surveys of thoracic surgery throughout Japan since 1986 to determine the statistics regarding the number of procedures according to operative category. Here, we have summarized the results from our annual survey of thoracic surgery performed during 2014.

Thoracic surgery was classified into three categories—cardiovascular, general thoracic, and esophageal surgery—and the patient data were examined and analyzed for each group. Access to the computerized data is offered to all members of this Association. We honor and value all member’s continued kind support and contributions (Tables 1, 2).

**Table 1:** **Table 1** Questionnaires sent out and received back by the end of December 2015

	Sent out	Returned	Response rate (%)
(A) Cardiovascular surgery	578	561	97.1
(B) General thoracic surgery	762	732	96.1
(C) Esophageal surgery	626	601	96.0

**Table 2:** **Table 2** Categories subclassified according to the number of operations performed

Number of operations performed	Category	
	Cardiovascular surgery	General thoracic surgery
0	21	30
1–24	42	81
25–49	86	108
50–99	157	202
100–149	103	137
150–199	52	80
≧200	100	94
Total	561	732

Number of operations performed	Esophageal surgery	
0	98	
1–4	145	
5–9	117	
10–19	108	
20–29	39	
30–39	27	
40–49	25	
≧50	42	
Total	601	

The incidence of hospital mortality was added to the survey to determine the nationwide status, which has contributed to the Japanese surgeons to understand the present status of thoracic surgery in Japan and to make progress to improve operative results by comparing their work with those of others. The Association was able to gain a better understanding of the present problems as well as the future prospects, which has been reflected to its activity including education of its members. Thirty-day mortality (so-called “operative mortality”) is defined as death within 30 days of operation regardless of the patient’s geographic location and even though the patient had been discharged from the hospital.

Hospital mortality is defined as death within any time interval after an operation if the patient had not been discharged from the hospital. Hospital-to-hospital transfer is not considered discharge in the categories of cardiovascular surgery and esophageal surgery: transfer to a nursing home or a rehabilitation unit is considered hospital discharge unless the patient subsequently dies of complications of the operation. While hospital-to-hospital transfer after 30 days of operation is considered discharge in the categories of general thoracic surgery, because data of national clinical database (NCD) 2014 were used in this category, and hospital-to-hospital transfer after 30 days of operation is considered discharge in NCD.

## Abstract of the survey

We sent out survey questionnaire forms to the departments of each category in all 1039 institutions (578 cardiovascular, 762 general thoracic, and 626 esophageal) nationwide in early April 2014. The response rates in each category by the end of December 2015 were 97.1, 96.1, and 96.0 %, respectively. This high response rate has been keep throughout recent survey, and more than 96 % response rate in all fields in 2014 survey has to be congratulated.

## 2014 Final report

### (A) Cardiovascular surgery

First, we are very pleased with the high response rate to our survey of cardiovascular surgery (97.1 %), which definitely enhances the quality of this annual report. We very much appreciate the enormous effort put into completing the survey at each participating institution.

**Fig. 1:**
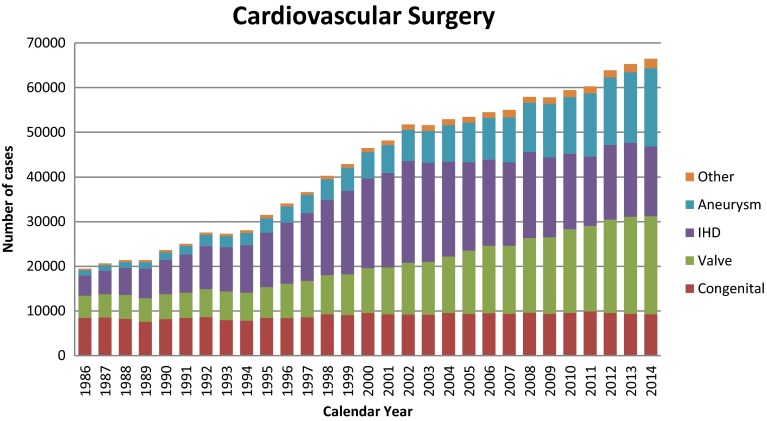
Cardiovascular surgery. *IHD* ischemic heart disease

Figure 1 shows the development of cardiovascular surgery in Japan over the last 28 years. Aneurysm surgery includes only operations for thoracic and thoracoabdominal aortic aneurysm. Pacemaker implantation includes only transthoracic implantation, and transvenous implantation is excluded. The number of pacemaker and assist device implantation operations is not included in the total number of surgical operations. A total of 66,453 cardiovascular operations were performed at 561 institutions during 2014 alone and included 30 heart transplantations, which were restarted in 1999.

The number of operations for congenital heart disease (9269 cases) decreased slightly (1.0 %) compared with that of 2013 (9366 cases), and 2.9 % decrease when compared with the data of 10 years ago (9545 cases in 2004). The number of operations for adult cardiac disease (21,939 cases in valvular heart disease, 17,498 cases in thoracic aortic aneurysm, and 2118 cases for other procedures) increased compared with those of 2013 (0.8, 11.0, and 13.2 %, respectively) except for ischemic heart disease (15,629 cases), which decreased 5.6 % of that in 2013. During the last 10 years, the numbers of operations for adult heart disease increased constantly except for that for ischemic heart disease (73.8 % increase in valvular heart disease, 26.5 % decrease in ischemic heart disease, 114.5 % increase in thoracic aortic aneurysm, and 56.5 % increase in other procedures compared those of 2004). The concomitant coronary artery bypass grafting procedure (CABG) is not included in ischemic heart disease but included in other categories, such as valvular heart disease and thoracic aneurysm in our study, and then, the number of CABG still remained over 20,000 cases per year (20,991 cases) in 2014.

Data for individual categories are summarized in tables through 3, 4, 5, 6, 7, 8 and 9.

**Table 3:** **Table 3** Congenital (total; 9269) (1) CPB (+) (total; 6894)

		Neonate				Infant				1–17 years				≧18 years				Total			
		Cases	30-day mortality		Hospital mortality	Cases	30-day mortality		Hospital mortality	Cases	30-day mortality		Hospital mortality	Cases	30-day mortality		Hospital mortality	Cases	30-day mortality		Hospital mortality
			Hospital	After discharge			Hospital	After discharge			Hospital	After discharge			Hospital	After discharge			Hospital	After discharge	
1	PDA	1	1 (100.0)	0	1 (100.0)	2	0	0	0	1	0	0	0	13	0	0	0	17	1 (5.9)	0	1 (5.9)
2	Coarctation (simple)	11	0	0	0	10	0	0	0	11	0	0	0	7	0	0	0	39	0	0	0
3	+VSD	39	0	0	0	51	0	0	0	12	0	0	0	6	0	0	0	108	0	0	0
4	+DORV	8	0	0	0	12	0	0	1 (8.3)	3	0	0	0	0	0	0	0	23	0	0	1 (4.3)
5	+AVSD	1	0	0	0	3	1 (33.3)	0	1 (33.3)	3	0	0	0	1	0	0	0	8	1 (12.5)	0	1 (12.5)
6	+TGA	9	0	0	1 (11.1)	4	0	0	0	3	0	0	0	4	0	0	0	20	0	0	1 (5.0)
7	+SV	4	1 (25.0)	0	1 (25.0)	8	0	0	0	2	0	0	0	1	0	0	0	15	1 (6.7)	0	1 (6.7)
8	+Others	3	1 (33.3)	0	1 (33.3)	8	0	0	0	3	0	0	0	1	0	0	0	15	1 (6.7)	0	1 (6.7)
9	Interrupt. of Ao (simple)	4	0	0	0	1	0	0	0	1	0	0	0	0	0	0	0	6	0	0	0
10	+VSD	33	1 (3.0)	0	3 (9.1)	23	1 (4.3)	0	2 (8.7)	9	0	0	0	5	0	0	0	70	2 (2.9)	0	5 (7.1)
11	+DORV	1	0	0	0	3	0	0	0	0	0	0	0	0	0	0	0	4	0	0	0
12	+Truncus	0	0	0	0	0	0	0	0	0	0	0	0	0	0	0	0	0	0	0	0
13	+TGA	1	1 (100.0)	0	1 (100.0)	0	0	0	0	12	0	0	0	0	0	0	0	13	1 (7.7)	0	1 (7.7)
14	+Others	5	0	0	0	3	0	0	0	2	0	0	1 (50.0)	0	0	0	0	10	0	0	1 (10.0)
15	Vascular ring	0	0	0	0	8	0	0	0	2	0	0	0	0	0	0	0	10	0	0	0
16	PS	1	0	0	0	10	0	0	0	19	0	0	0	9	1 (11.1)	0	1 (11.1)	39	1 (2.6)	0	1 (2.6)
17	PAIVS or critical PS	18	0	0	1 (5.6)	44	1 (2.3)	0	1 (2.3)	84	0	0	0	6	0	0	0	152	1 (0.7)	0	2 (1.3)
18	TAPVR	113	8 (7.1)	0	15 (13.3)	65	4 (6.2)	0	6 (9.2)	5	0	0	0	0	0	0	0	183	12 (6.6)	0	21 (11.5)
19	PAPVR ± ASD	0	0	0	0	5	0	0	0	45	0	0	1 (2.2)	27	1 (3.7)	0	1 (3.7)	77	1 (1.3)	0	2 (2.6)
20	ASD	20	0	0	0	67	0	0	1 (1.5)	667	0	0	1 (0.1)	494	0	0	0	1248	0	0	2 (0.2)
21	Cor triatriatum	2	0	0	0	14	1 (7.1)	0	1 (7.1)	11	0	0	0	4	0	0	0	31	1 (3.2)	0	1 (3.2)
22	AVSD (partial)	1	0	0	0	7	0	0	0	33	1 (3.0)	0	1 (3.0)	24	0	0	0	65	1 (1.5)	0	1 (1.5)
23	AVSD (complete)	2	0	0	0	108	0	1 (0.9)	2 (1.9)	67	0	0	0	4	0	0	1 (25.0)	181	0	1 (0.6)	3 (1.7)
24	+TOF or DORV	1	0	0	0	11	1 (9.1)	0	1 (9.1)	15	3 (20.0)	0	3 (20.0)	0	0	0	0	27	4 (14.8)	0	4 (14.8)
25	+Others	3	0	0	0	4	0	0	1 (25.0)	6	0	0	0	9	0	0	0	22	0	0	1 (4.5)
26	VSD (subarterial)	4	1 (25.0)	0	1 (25.0)	100	0	0	0	199	0	0	0	33	0	0	0	336	1 (0.3)	0	1 (0.3)
27	VSD (perimemb./muscular)	9	0	0	0	742	3 (0.4)	0	4 (0.5)	353	2 (0.6)	1 (0.3)	2 (0.6)	71	0	0	0	1175	5 (0.4)	1 (0.1)	6 (0.5)
28	VSD + PS	0	0	0	0	15	0	0	0	20	0	0	0	3	0	0	0	38	0	0	0
29	DCRV ± VSD	1	0	0	0	16	0	0	0	28	0	0	0	20	0	0	0	65	0	0	0
30	Aneurysm of sinus valsalva	0	0	0	0	1	0	0	0	8	0	0	0	22	0	0	0	31	0	0	0
31	TOF	9	0	0	0	176	1 (0.6)	0	2 (1.1)	212	0	0	1 (0.5)	42	1 (2.4)	0	2 (4.8)	439	2 (0.5)	0	5 (1.1)
32	PA + VSD	4	0	0	0	49	1 (2.0)	0	3 (6.1)	92	0	0	1 (1.1)	7	0	0	0	152	1 (0.7)	0	4 (2.6)
33	DORV	17	0	0	0	106	3 (2.8)	0	3 (2.8)	108	0	0	2 (1.9)	11	1 (9.1)	0	1 (9.1)	242	4 (1.7)	0	6 (2.5)
34	TGA (simple)	102	8 (7.8)	1 (1.0)	8 (7.8)	13	0	0	0	5	0	0	0	1	0	0	0	121	8 (6.6)	1 (0.8)	8 (6.6)
35	+VSD	31	1 (3.2)	0	1 (3.2)	12	1 (8.3)	0	1 (8.3)	8	0	0	0	0	0	0	0	51	2 (3.9)	0	2 (3.9)
36	VSD + PS	1	0	0	0	11	0	0	1 (9.1)	15	1 (6.7)	0	1 (6.7)	6	0	0	0	33	1 (3.0)	0	2 (6.1)
37	Corrected TGA	3	0	0	0	23	0	0	0	40	1 (2.5)	0	2 (5.0)	13	0	0	0	79	1 (1.3)	0	2 (2.5)
38	Truncus arteriosus	11	1 (9.1)	0	1 (9.1)	22	0	0	1 (4.5)	14	0	0	0	2	0	0	0	49	1 (2.0)	0	2 (4.1)
39	SV	22	2 (9.1)	0	7 (31.8)	202	4 (2.0)	0	6 (3.0)	263	4 (1.5)	1 (0.4)	8 (3.0)	20	1 (5.0)	0	1 (5.0)	507	11 (2.2)	1 (0.2)	22 (4.3)
40	TA	5	0	0	0	44	1 (2.3)	0	1 (2.3)	53	0	0	1 (1.9)	10	0	0	0	112	1 (0.9)	0	2 (1.8)
41	HLHS	40	2 (5.0)	0	5 (12.5)	124	9 (7.3)	0	15 (12.1)	60	1 (1.7)	0	2 (3.3)	0	0	0	0	224	12 (5.4)	0	22 (9.8)
42	Aortic valve lesion	6	0	0	0	14	1 (7.1)	0	1 (7.1)	89	1 (1.1)	0	1 (1.1)	16	1 (6.3)	0	1 (6.3)	125	3 (2.4)	0	3 (2.4)
43	Mitral valve lesion	2	0	0	0	28	1 (3.6)	0	1 (3.6)	72	1 (1.4)	0	2 (2.8)	8	0	0	0	110	2 (1.8)	0	3 (2.7)
44	Ebstein	15	0	0	3 (20.0)	14	0	0	1 (7.1)	34	0	0	0	16	2 (12.5)	0	2 (12.5)	79	2 (2.5)	0	6 (7.6)
45	Coronary disease	1	0	0	0	8	0	0	0	14	1 (7.1)	0	1 (7.1)	22	0	0	0	45	1 (2.2)	0	1 (2.2)
46	Others	21	0	0	1 (4.8)	46	1 (2.2)	0	3 (6.5)	35	0	0	0	9	0	0	1 (11.1)	111	1 (0.9)	0	5 (4.5)
47	Redo VSD	0	0	0	0	5	0	0	0	13	0	0	0	3	0	0	0	21	0	0	0
48	PS release	0	0	0	0	10	0	0	0	52	0	0	0	24	0	0	0	86	0	0	0
49	RV-PA conduit replace	0	0	0	0	4	0	0	0	54	0	0	0	37	0	0	0	95	0	0	0
50	Others	4	0	0	1 (25.0)	41	2 (4.9)	0	3 (7.3)	97	0	0	0	43	0	0	0	185	2 (1.1)	0	4 (2.2)
Total		589	28 (4.8)	1 (0.2)	52 (8.8)	2297	37 (1.6)	1 (0.04)	63 (2.7)	2,954	16 (0.5)	2 (0.1)	31 (1.0)	1054	8 (0.8)	0	11 (1.0)	6894	89 (1.3)	4 (0.1)	157 (2.3)

Values in parenthesis represent mortality %

*CPB* cardiopulmonary bypass, *PDA* patient ductus arteriosus, *VSD* ventricular septal defect, *DORV* double outlet right ventricle, *AVSD* atrioventricular septal defect, *TGA* transposition of great arteries, *SV* single ventricle, Interupt. of Ao. interruption of aorta, *PS* pulmonary stenosis, *PA-IVS* pulmonary atresia with intact ventricular septum, *TAPVR* total anomalous pulmonary venous return, *PAPVR* partial anomalous pulmonary venous return, *ASD* atrial septal defect, *TOF* tetralogy of Fallot, DCRV double-chambered right ventricle, *TA* tricuspid atresia, *HLHS* hypoplastic left heart syndrome, *RV-PA* right ventricle-pulmonary artery

**Table 4:** (2) CPB (−) (total; 2375)

		Neonate				Infant				1–17 years				≧18 years				Total			
		Cases	30-day mortality		Hospital mortality	Cases	30-day mortality		Hospital mortality	Cases	30-day mortality		Hospital mortality	Cases	30-day mortality		Hospital mortality	Cases	30-day mortality		Hospital mortality
			Hospital	After discharge			Hospital	After discharge			Hospital	After discharge			Hospital	After discharge			Hospital	After discharge	
1	PDA	430	4 (0.9)	0	5 (1.2)	230	2 (0.9)	0	4 (1.7)	40	0	0	0	2	0	0	0	702	6 (0.9)	0	9 (1.3)
2	Coarctation (simple)	21	1 (4.8)	0	1 (4.8)	27	0	0	0	4	0	0	0	0	0	0	0	52	1 (1.9)	0	1 (1.9)
3	+VSD	39	0	0	0	16	0	0	0	3	0	0	0	1	0	0	0	59	0	0	0
4	+DORV	9	0	0	0	9	0	0	0	0	0	0	0	0	0	0	0	18	0	0	0
5	+AVSD	2	0	0	0	1	0	0	0	4	0	0	0	0	0	0	0	7	0	0	0
6	+TGA	4	0	0	0	1	0	0	0	2	0	0	0	1	0	0	0	8	0	0	0
7	+SV	12	0	0	1 (8.3)	8	0	0	0	0	0	0	0	0	0	0	0	20	0	0	1 (5.0)
8	+Others	8	0	0	0	2	0	0	0	0	0	0	0	0	0	0	0	10	0	0	0
9	Interrupt. of Ao (simple)	2	0	0	0	1	0	0	0	1	0	0	0	1	0	0	0	5	0	0	0
10	+VSD	25	0	0	0	8	0	0	0	1	0	0	0	1	0	0	0	35	0	0	0
11	+DORV	3	0	0	0	1	0	0	0	0	0	0	0	0	0	0	0	4	0	0	0
12	+Truncus	1	0	0	0	0	0	0	0	0	0	0	0	0	0	0	0	1	0	0	0
13	+TGA	1	0	0	1 (100.0)	0	0	0	0	0	0	0	0	1	0	0	0	2	0	0	1 (50.0)
14	+Others	1	0	0	0	1	0	0	0	0	0	0	0	0	0	0	0	2	0	0	0
15	Vascular ring	5	0	0	0	8	0	0	0	7	0	0	0	0	0	0	0	20	0	0	0
16	PS	1	0	0	0	0	0	0	0	3	0	0	0	0	0	0	0	4	0	0	0
17	PAIVS or critical PS	25	1 (4.0)	0	1 (4.0)	20	0	0	1 (5.0)	8	1 (12.5)	0	2 (25.0)	0	0	0	0	53	2 (3.8)	0	4 (7.5)
18	TAPVR	3	0	0	0	8	2 (25.0)	0	2 (25.0)	1	0	0	0	1	0	0	0	13	2 (15.4)	0	2 (15.4)
19	PAPVR ± ASD	0	0	0	0	0	0	0	0	1	0	0	0	1	0	0	0	2	0	0	0
20	ASD	0	0	0	0	0	0	0	0	0	0	0	0	0	0	0	0	0	0	0	0
21	Cor triatriatum	0	0	0	0	0	0	0	0	2	0	0	0	0	0	0	0	2	0	0	0
22	AVSD (partial)	1	0	0	0	3	0	0	0	2	0	0	0	1	0	0	0	7	0	0	0
23	AVSD (complete)	35	0	0	1 (2.9)	72	0	0	0	3	0	0	0	0	0	0	0	110	0	0	1 (0.9)
24	+TOF or DORV	2	0	0	0	7	0	0	0	4	0	0	0	0	0	0	0	13	0	0	0
25	+Others	7	0	0	0	1	0	0	0	0	0	0	0	0	0	0	0	8	0	0	0
26	VSD (subarterial)	1	0	0	0	8	0	0	0	1	0	0	0	0	0	0	0	10	0	0	0
27	VSD (perimemb./muscular)	49	0	0	2 (4.1)	107	0	0	2 (1.9)	2	0	0	0	2	0	0	0	160	0	0	4 (2.5)
28	VSD + PS	1	0	0	0	0	0	0	0	0	0	0	0	0	0	0	0	1	0	0	0
29	DCRV ± VSD	1	0	0	0	1	0	0	0	0	0	0	0	1	0	0	0	3	0	0	0
30	Aneurysm of sinus valsalva	0	0	0	0	0	0	0	0	0	0	0	0	0	0	0	0	0	0	0	0
31	TOF	28	2 (7.1)	0	2 (7.1)	88	0	0	0	7	0	0	0	1	0	0	0	124	2 (1.6)	0	2 (1.6)
32	PA + VSD	23	0	0	0	69	1 (1.4)	0	1 (1.4)	23	1 (4.3)	0	1 (4.3)	0	0	0	0	115	2 (1.7)	0	2 (1.7)
33	DORV	36	0	0	0	55	0	0	0	14	0	0	0	0	0	0	0	105	0	0	0
34	TGA (simple)	6	0	0	0	6	0	0	0	0	0	0	0	0	0	0	0	12	0	0	0
35	+VSD	7	0	0	0	4	0	0	0	3	0	0	0	0	0	0	0	14	0	0	0
36	VSD + PS	12	0	0	0	14	0	0	0	1	0	0	0	13	0	0	0	40	0	0	0
37	Corrected TGA	8	0	0	0	26	0	0	0	8	0	0	0	8	0	0	0	50	0	0	0
38	Truncus arteriosus	15	1 (6.7)	0	1 (6.7)	5	0	0	0	5	0	0	0	0	0	0	0	25	1 (4.0)	0	1 (4.0)
39	SV	73	2 (2.7)	0	4 (5.5)	58	2 (3.4)	0	3 (5.2)	24	0	0	0	6	0	0	0	161	4 (2.5)	0	7 (4.3)
40	TA	25	0	0	0	13	1 (7.7)	0	1 (7.7)	9	0	0	0	0	0	0	0	47	1 (2.1)	0	1 (2.1)
41	HLHS	97	3 (3.1)	0	7 (7.2)	14	0	0	0	16	0	0	2 (12.5)	1	0	0	0	128	3 (2.3)	0	9 (7.0)
42	Aortic valve lesion	1	0	0	0	2	0	0	0	1	0	0	0	0	0	0	0	4	0	0	0
43	Mitral valve lesion	2	0	0	0	0	0	0	0	0	0	0	0	0	0	0	0	2	0	0	0
44	Ebstein	6	0	0	0	1	0	0	0	3	0	0	0	1	0	0	0	11	0	0	0
45	Coronary disease	1	0	0	0	2	1 (50.0)	0	1 (50.0)	4	0	0	0	0	0	0	0	7	1 (14.3)	0	1 (14.3)
46	Others	15	1 (6.7)	0	1 (6.7)	52	0	0	0	56	0	0	0	18	0	0	0	141	1 (0.7)	0	1 (0.7)
47	Redo VSD	0	0	0	0	0	0	0	0	0	0	0	0	0	0	0	0	0	0	0	0
48	PS release	1	0	0	1 (100.0)	2	0	0	0	1	0	0	0	0	0	0	0	4	0	0	1 (25.0)
49	RV-PA conduit replace	0	0	0	0	0	0	0	0	0	0	0	0	0	0	0	0	0	0	0	0
50	Others	6	0	0	0	22	0	0	1 (4.5)	21	1 (4.8)	0	1 (4.8)	5	0	0	0	54	1 (1.9)	0	2 (3.7)
Total		1051	15 (1.4)	0	28 (2.7)	973	9 (0.9)	0	16 (1.6)	285	3 (1.1)	0	6 (2.1)	66	0	0	0	2375	27 (1.1)	0	50 (2.1)

Values in parenthesis represent mortality %

*CPB* cardiopulmonary bypass, *PDA* patient ductus arteriosus, *VSD* ventricular septal defect, *DORV *double outlet right ventricle, *AVSD* atrioventricular septal defect, *TGA* transposition of great arteries, *SV* single ventricle, Interupt. of Ao. interruption of aorta, *PS* pulmonary stenosis, *PA-IVS* pulmonary atresia with intact ventricular septum, *TAPVR* total anomalous pulmonary venous return, *PAPVR* partial anomalous pulmonary venous return, *ASD* atrial septal defect, *TOF* tetralogy of Fallot, *DCRV* double-chambered right ventricle, *TA* tricuspid atresia, *HLHS* hypoplastic left heart syndrome, *RV-PA* right ventricle-pulmonary artery

**Table 5:** (3) Main procedure

		Neonate				Infant				1–17 years				≧18 years				Total			
		Cases	30-day mortality		Hospital mortality	Cases	30-day mortality		Hospital mortality	Cases	30-day mortality		Hospital mortality	Cases	30-day mortality		Hospital mortality	Cases	30-day mortality		Hospital mortality
			Hospital	After discharge			Hospital	After discharge			Hospital	After discharge			Hospital	After discharge			Hospital	After discharge	
1	SP shunt	149	4 (2.7)	0	8 (5.4)	357	6 (1.7)	0	9 (2.5)	42	0	0	1 (2.4)	1	0	0	0	549	10 (1.8)	0	18 (3.3)
2	PAB	387	7 (1.8)	0	15 (3.9)	263	2 (0.8)	0	5 (1.9)	11	0	0	0	0	0	0	0	661	9 (1.4)	0	20 (3.0)
3	Bidirectional Glenn or hemi-Fontan ± α	1	0	0	0	240	2 (0.8)	0	2 (0.8)	106	1 (0.9)	0	1 (0.9)	4	0	0	0	351	3 (0.9)	0	3 (0.9)
4	Damus–Kaye–Stansel operation	2	0	0	0	36	1 (2.8)	0	1 (2.8)	15	0	0	0	3	0	0	1 (33.3)	56	1 (1.8)	0	2 (3.6)
5	PA reconstruction/repair (including redo)	16	0	0	1 (6.3)	106	2 (1.9)	0	3 (2.8)	140	0	0	2 (1.4)	25	0	0	0	287	2 (0.7)	0	6 (2.1)
6	RVOT reconstruction/repair	12	0	0	0	111	1 (0.9)	0	2 (1.8)	202	1 (0.5)	0	3 (1.5)	53	0	0	1 (1.9)	378	2 (0.5)	0	6 (1.6)
7	Rastelli procedure	9	0	0	0	39	0	0	1 (2.6)	94	0	0	2 (2.1)	11	0	0	0	153	0	0	3 (2.0)
8	Arterial switch procedure	134	9 (6.7)	1 (0.7)	9 (6.7)	40	1 (2.5)	0	1 (2.5)	2	1 (50.0)	0	2 (100.0)	0	0	0	0	176	11 (6.3)	1 (0.6)	12 (6.8)
9	Atrial switch procedure	2	1 (50.0)	0	1 (50.0)	4	0	0	0	1	0	0	0	0	0	0	0	7	1 (14.3)	0	1 (14.3)
10	Double switch procedure	0	0	0	0	0	0	0	0	15	0	0	0	0	0	0	0	15	0	0	0
11	Repair of anomalous origin of CA	1	0	0	0	4	0	0	0	8	0	0	0	4	0	0	0	17	0	0	0
12	Closure of coronary AV fistula	1	0	0	0	5	0	0	0	4	0	0	0	29	0	0	0	39	0	0	0
13	Fontan/TCPC	0	0	0	0	5	0	0	0	362	1 (0.3)	0	4 (1.1)	30	0	0	0	397	1 (0.3)	0	4 (1.0)
14	Norwood procedure	29	0	0	2 (6.9)	93	8 (8.6)	0	17 (18.3)	2	0	0	0	1	0	0	0	125	8 (6.4)	0	19 (15.2)
15	Ventricular septation	0	0	0	0	10	0	0	0	4	0	0	0	1	0	0	0	15	0	0	0
16	Left side AV valve repair (including Redo)	3	1 (33.3)	0	2 (66.7)	45	2 (4.4)	0	2 (4.4)	71	0	0	0	28	0	0	0	147	3 (2.0)	0	4 (2.7)
17	Left side AV valve replace (including Redo)	0	0	0	0	9	0	0	0	37	2 (5.4)	0	2 (5.4)	19	0	0	0	65	2 (3.1)	0	2 (3.1)
18	Right side AV valve repair (including Redo)	4	0	0	2 (50.0)	14	0	0	1 (7.1)	34	0	0	0	38	0	0	0	90	0	0	3 (3.3)
19	Right side AV valve replace (including Redo)	0	0	0	0	2	1 (50.0)	0	1 (50.0)	9	0	0	0	15	2 (13.3)	0	2 (13.3)	26	3 (11.5)	0	3 (11.5)
20	Common AV valve repair (including Redo)	2	0	0	0	33	1 (3.0)	0	3 (9.1)	34	2 (5.9)	0	2 (5.9)	1	0	0	0	70	3 (4.3)	0	5 (7.1)
21	Common AV valve replace (including Redo)	1	0	0	0	2	0	0	0	7	0	0	1 (14.3)	1	0	0	0	11	0	0	1 (9.1)
22	Repair of supra-aortic stenosis	3	0	0	0	9	0	0	0	15	0	0	0	2	0	0	0	29	0	0	0
23	Repair of subaortic stenosis (including Redo)	2	0	0	2 (100.0)	7	0	0	0	24	2 (8.3)	0	2 (8.3)	7	0	0	0	40	2 (5.0)	0	4 (10.0)
24	Aortic valve plasty ± VSD closure	4	0	0	0	8	0	0	0	31	0	0	0	2	0	0	0	45	0	0	0
25	Aortic valve replacement	0	0	0	0	0	0	0	0	19	0	0	0	23	0	0	0	42	0	0	0
26	AVR with annular enlargement	0	0	0	0	0	0	0	0	12	0	0	0	3	1 (33.3)	0	1 (33.3)	15	1 (6.7)	0	1 (6.7)
27	Aortic root replace (except Ross)	0	0	0	0	1	0	0	0	10	0	0	0	5	0	0	0	16	0	0	0
28	Ross procedure	0	0	0	0	0	0	0	0	11	1 (9.1)	0	1 (9.1)	2	0	0	0	13	1 (7.7)	0	1 (7.7)
Total		762	22 (2.9)	1 (0.1)	42 (5.5)	1443	27 (1.9)	0	48 (3.3)	1322	11 (0.8)	0	23 (1.7)	308	3 (1.0)	0	5 (1.6)	3835	63 (1.6)	1 (0.03)	118 (3.1)

Values in parenthesis represent mortality %

*SP* systemic-pulmonary, *PAB* pulmonary artery banding, *PA* pulmonary artery, *RVOT* right ventricular outflow tract, *CA* coronary artery, *AV* fistula arteriovenous fistula, *TCPC* total cavopulmonary connection, *AV* valve atrioventricular valve, *VSD* ventricular septal defect, *AVR* aortic valve replacement

**Table 6:** **Table 4** Acquired (total, (1) + (2) + (4) + (5) + (6) + (7) + isolated ope. for arrhythmia in (3); 39,485 (1) Valvular heart disease (total; 21,939)

	Valve	Cases	Operation					30-day mortality				Hospital mortality		Redo			
			Mechanical	Bioprosthesis	Ross procedure	Repair	With CABG	Hospital		After discharge				Cases	30-day mortality		Hospital mortality
								Replace	Repair	Replace	Repair	Replace	Repair		Hospital	After discharge	
Isolated	A	10,219	1884	8037	1	297	2298	156 (1.6)	5 (1.7)	3 (0.03)	0	238 (2.4)	9 (3.0)	371	20 (5.4)	0	35 (9.4)
	M	4851	684	918		3249	716	56 (3.5)	16 (0.5)	2 (0.1)	0	95 (5.9)	35 (1.1)	344	10 (2.9)	0	27 (7.8)
	T	253	10	68		175	25	5 (6.4)	5 (2.9)	0	0	9 (11.5)	7 (4.0)	48	3 (6.3)	0	6 (12.5)
	P	13	2	9		2	0	0	0	0	0	0	0	4	0	0	0
A + M	A	1537	388	1085	0	55	238	75 (4.9)		0		112 (7.3)		91	13 (14.3)	0	16 (17.6)
	M		275	422	0	832											
A + T	A	448	96	339	1	6	63	11 (2.5)		0		23 (5.1)		42	2 (4.8)	0	4 (9.5)
	T		3	5	0	435											
M + T	M	3513	494	1044		1972	313	53 (1.5)		0		94 (2.7)		234	13 (5.6)	0	22 (9.4)
	T		12	70		3424											
A + M + T	A	1056	255	759	0	39	130	39 (3.7)		0		64 (6.1)		66	8 (12.1)	0	11 (16.7)
	M		198	381	0	474											
	T		4	17	0	1032											
Others		49	5	22	0	14	2	1 (2.0)		0		2 (0.2)		10	0	0	0
Total		21,939	4310	13,176	2	12,006	3785	422 (1.9)		5 (0.02)		688 (3.1)		1210	69 (5.7)	0	121 (10.0)

Number of redo cases is included in total case number of 21,939

Values in parenthesis represent mortality %

*CABG* coronary artery bypass grafting,* A* aortic valve,* M* mitral valve,* T* tricuspid valve,* P* pulmonary valve

**Table 7:** 

	Cases	30-day mortality		Hospital mortality
		Hospital	After discharge	
TAVR	877	11	1	17

**Table 8:** (2) Ischemic heart disease (total, (A) + (B) + (C); 15,629) (*A*)* Isolated CABG* (*total*; (*a*)+(*b*);* 14,454*) (a-1) on-pump arrest CABG (total; 3277)

	Primary, elective				Primary, emergency				Redo, elective				Redo, emergency				Arterial graft only	Artery graft + SVG	SVG only	Others	Unclear
	Cases	30-day mortality		Hospital mortality	Cases	30-day mortality		Hospital mortality	Cases	30-day mortality		Hospital mortality	Cases	30-day mortality		Hospital mortality					
		Hospital	After discharge			Hospital	After discharge			Hospital	After discharge			Hospital	After discharge						
1VD	93	1 (1.1)	0	1 (1.1)	19	2 (10.5)	0	3 (15.8)	5	0	0	1 (20.0)	2	1 (50.0)	0	2 (100.0)	55	18	46	0	0
2VD	461	3 (0.7)	0	6 (1.3)	47	2 (4.3)	0	2 (4.3)	7	0	0	0	0	0	0	0	95	392	27	0	1
3VD	1512	10 (0.7)	0	12 (0.8)	161	15 (9.3)	0	19 (11.8)	15	1 (6.7)	0	2 (13.3)	1	1 (100.0)	0	1 (100.0)	92	1569	19	0	9
LMT	761	8 (1.1)	0	12 (1.6)	190	11 (5.8)	0	15 (7.9)	3	1 (33.3)	0	1 (33.3)	0	0	0	0	107	814	33	1	1
Total	2827	22 (0.8)	0	31 (1.1)	417	30 (7.2)	0	39 (9.4)	30	2 (6.7)	0	4 (13.3)	3	2 (66.7)	0	3 (100.0)	349	2793	125	1	11
Kawasaki	7	0	0	0	1	0	0	0	1	0	0	0	0	0	0	0	7	2	0	0	0
Hemodialysis	172	6 (3.5)	0	8 (4.7)	28	3 (10.7)	0	5 (17.9)	1	0	0	1 (100.0)	0	0	0	0	10	173	10	0	8

Values in parenthesis represent mortality %

*CABG* coronary artery bypass grafting, *1VD* one-vessel disease, *2VD* two-vessel disease, *3VD* three-vessel disease, *LMT* left main trunk, *SVG* saphenous vein graft, *LMT* includes LMT alone or LMT with other branch diseases

**Table 9:** (a-2) on-pump beating CABG (total; 2171)

	Primary, elective				Primary, emergency				Redo, elective				Redo, emergency				Arterial graft only	Artery graft + SVG	SVG only	Others	Unclear
	Cases	30-day mortality		Hospital mortality	Cases	30-day mortality		Hospital mortality	Cases	30-day mortality		Hospital mortality	Cases	30-day mortality		Hospital mortality					
		Hospital	After discharge			Hospital	After discharge			Hospital	After discharge			Hospital	After discharge						
1VD	35	2 (5.7)	0	2 (5.7)	31	2 (6.5)	0	5 (16.1)	4	0	0	1 (25.0)	3	1 (33.3)	0	1 (33.3)	40	6	25	0	2
2VD	255	4 (1.6)	0	6 (2.4)	51	4 (7.8)	0	6 (11.8)	11	1 (9.1)	0	1 (9.1)	6	2 (33.3)	0	4 (66.7)	74	224	18	0	7
3VD	894	15 (1.7)	0	28 (3.1)	170	15 (8.8)	0	18 (10.6)	7	0	0	0	1	1 (100.0)	0	1 (100.0)	118	918	24	0	12
LMT	479	6 (1.3)	0	8 (1.7)	216	24 (11.1)	0	33 (15.3)	6	0	0	0	2	1 (50.0)	0	1 (50.0)	103	564	31	0	5
Total	1663	27 (1.6)	0	44 (2.6)	468	45 (9.6)	0	62 (13.2)	28	1 (3.6)	0	2 (7.1)	12	5 (41.7)	0	7 (58.3)	335	1712	98	0	26
Kawasaki	2	0	0	0	1	1 (100.0)	0	1 (100.0)	0	0	0	0	1	0	0	0	1	1	0	0	2
Hemodialysis	139	4 (2.9)	0	11 (7.9)	30	3 (10.0)	0	4 (13.3)	6	0	0	0	2	0	0	1 (50.0)	17	142	10	0	8

Values in parenthesis represent mortality %

*CABG* coronary artery bypass grafting, *1VD* one-vessel disease, *2VD* two-vessel disease, *3VD* three-vessel disease, *LMT* left main trunk, *SVG* saphenous vein graft, *LMT* includes LMT alone or LMT with other branch diseases

**Table 10:** (b) off-pump CABG (total; 9006) (The present section also includes cases of planned off-pump CABG in which, during surgery, the change is made to an on-pump CABG or on-pump beating-heart procedure)

	Primary, elective				Primary, emergency				Redo, elective				Redo, emergency				Arterial graft only	Artery graft + SVG	SVG only	Others	Unclear
	Cases	30-day mortality		Hospital mortality	Cases	30-day mortality		Hospital mortality	Cases	30-day mortality		Hospital mortality	Cases	30-day mortality		Hospital mortality					
		Hospital	After discharge			Hospital	After discharge			Hospital	After discharge			Hospital	After discharge						
1VD	556	3 (0.5)	0	6 (1.1)	70	4 (5.7)	0	5 (7.1)	27	1 (3.7)	0	2 (7.4)	2	1 (50.0)	0	1 (50.0)	531	36	64	0	24
2VD	1446	10 (0.7)	1 (0.07)	13 (0.9)	144	8 (5.6)	0	9 (6.3)	12	0	0	0	2	0	0	0	521	977	56	0	50
3VD	3679	27 (0.7)	0	43 (1.2)	386	13 (3.4)	0	16 (4.1)	22	0	0	0	3	0	0	0	708	3237	41	0	104
LMT	2164	14 (0.6)	0	23 (1.1)	474	18 (3.8)	0	23 (4.9)	14	0	0	0	5	0	0	0	654	1934	45	0	24
Total	7845	54 (0.7)	1 (0.01)	85 (1.1)	1074	43 (4.0)	0	53 (4.9)	75	1 (1.3)	0	2 (2.7)	12	1 (8.3)	0	1 (8.3)	2414	6184	206	0	202
Kawasaki	16	0	0	0	0	0	0	0	0	0	0	0	0	0	0	0	13	2	1	0	0
Hemodialysis	524	4 (0.8)	0	9 (1.7)	79	4 (5.1)	0	4 (5.1)	7	0	0	0	1	0	0	0	99	453	13	0	46

Values in parenthesis represent mortality %

*CABG* coronary artery bypass grafting, *1VD* one-vessel disease, *2VD* two-vessel disease, *3VD* three-vessel disease, *LMT* left main trunk, *SVG* saphenous vein graft, *LMT* includes LMT alone or LMT with other branch diseases

**Table 11:** (c) Includes cases of conversion, during surgery, from off-pump CABG to on-pump CABG or on-pump beating-heart CABG (total; 156)

	Primary, elective				Primary, emergency				Redo, elective				Redo, emergency			
	Cases	30-day mortality		Hospital mortality	Cases	30-day mortality		Hospital mortality	Cases	30-day mortality		Hospital mortality	Cases	30-day mortality		Hospital mortality
		Hospital	After discharge			Hospital	After discharge			Hospital	After discharge			Hospital	After discharge	
A conversion to on-pump CABG arrest heart	27	1 (3.7)	0	0	3	0	0	0	0	0	0	0	0	0	0	0
A conversion to on-pump beating-heart CABG	100	4 (4.0)	0	4 (4.0)	26	3 (11.5)	0	3 (11.5)	0	0	0	0	0	0	0	0
Total	127	5 (3.9)	0	4 (3.1)	29	3 (10.3)	0	3 (10.3)	0	0	0	0	0	0	0	0
Hemodialysis	15	1 (6.7)	0	1 (6.7)	1	0	0	0	0	0	0	0	0	0	0	0

Values in parenthesis represent mortality %

*CABG* coronary artery bypass grafting

**Table 12:** (*B*)* Operation for complications of MI* (*total*;* 1175*)

	Chronic				Acute				Concomitant operation		
	Cases	30-day mortality		Hospital mortality	Cases	30-day mortality		Hospital mortality			
		Hospital	After discharge			Hospital	After discharge		CABG	MVP	MVR
Infarctectomy or aneurysmectomy	257	6 (2.3)	0	13 (5.1)	38	6 (15.8)	0	7 (18.4)	164	59	19
VSP closure	51	4 (7.8)	0	5 (9.8)	245	70 (28.6)	0	82 (33.5)	77	1	7
Cardiac rupture	21	1 (4.8)	0	5 (23.8)	199	73 (36.7)	0	78 (39.2)	23	1	1
Mitral regurgitation											
1) Papillary muscle rupture	10	1 (10.0)	0	1 (10.0)	46	10 (21.7)	1 (2.2)	12 (26.1)	18	11	46
2) Ischemic	251	7 (2.8)	0	17 (6.8)	27	7 (25.9)	0	7 (25.9)	221	174	53
Others	19	0	0	0	11	1 (9.1)	0	3 (27.3)	3	4	0
Total	609	19 (3.1)	0	41 (6.7)	566	167 (29.5)	1 (0.2)	189 (33.4)	506	250	126

Values in parenthesis represent mortality %

Acute, within 2 weeks from the onset of myocardial infarction

*MI* myocardial infarction,* CABG* coronary artery bypass grafting,* MVP* mitral valve repair,* MVR* mitral valve replacement,* VSP* ventricular septal perforation

**Table 13:** (*C*)* TMLR* (*total*;* 0*)

	Cases	30-day mortality		Hospital mortality
		Hospital	After discharge	
Isolated	0	0	0	0
With CABG	0	0	0	0
Total	0	0	0	0

*TMLR* transmyocardial laser revascularization

**Table 14:** (3) Operation for arrhythmia (total; 3855)

	Cases	30-day mortality		Hospital mortality	Concomitant operation						
					Isolated	Congenital	Valve	IHD	Others	Multiple combination	
		Hospital	After discharge							2 categories	3 categories
Maze	3486	34 (1.0)	0	55 (1.6)	15	127	3,162	375	216	440	32
For WPW	2	0	0	0	0	1	1	0	0	0	0
For ventricular tachyarrhythmia	35	2 (5.7)	0	3 (8.6)	2	3	14	13	5	2	0
Others	332	3 (0.9)	0	4 (1.2)	89	7	193	57	25	34	3
Total	3855	39 (1.0)	0	62 (1.6)	106	138	3370	445	246	476	35

Values in parenthesis represent mortality %. Except for 106 isolated cases, all remaining 3749 cases are doubly allocated, one for this subgroup and the other for the subgroup corresponding to the concomitant operations

*WPW* Wolff–Parkinson–White syndrome, *IHD* ischemic heart disease

**Table 15:** (4) Operation for constrictive pericarditis (total; 178)

	CPB (+)				CPB (−)			
	Cases	30-day mortality		Hospital mortality	Cases	30-day mortality		Hospital mortality
		Hospital	After discharge			Hospital	After discharge	
Total	102	12 (11.8)	0	15 (14.7)	76	0	0	5 (6.6)

Values in parenthesis represent mortality %

*CPB* cardiopulmonary bypass

**Table 16:** (5) Cardiac tumor (total; 602)

	Cases	30-day mortality		Hospital mortality	Concomitant operation			
		Hospital	After discharge		AVR	MVR	CABG	Others
Benign tumor	530	4 (0.8)	0	7 (1.3)	10	11	25	70
Cardiac myxoma	419	2 (0.5)	0	2 (0.5)	4	8	20	59
Papillary fibroelastoma	46	0	0	2 (4.3)	4	2	1	7
Rhabdomyoma	4	1 (25.0)	0	1 (25.0)	0	0	0	0
Others	61	1 (1.6)	0	2 (3.3)	2	1	4	4
Malignant tumor	72	4 (5.6)	1 (1.4)	11 (15.3)	2	3	2	11
Primary	45	2 (4.4)	0	3 (6.7)	2	3	1	7
Metastatic	27	2 (7.4)	1 (3.7)	8 (29.6)	0	0	1	4

Values in parenthesis represent mortality %

*AVR* aortic valve replacement,* MVR* mitral valve replacement,* CABG* coronary artery bypass grafting

**Table 17:** (6) HOCM and DCM (total; 211)

	Cases	30-day mortality		Hospital mortality	Concomitant operation			
		Hospital	After discharge		AVR	MVR	MVP	CABG
Myectomy	171	5 (2.9)	0	8 (4.7)	110	19	23	13
Myotomy	5	0	0	0	1	2	0	0
No-resection	14	1 (7.1)	0	1 (7.1)	2	5	16	0
Volume reduction surgery of the left ventricle	21	3 (14.3)	0	4 (19.0)	0	6	6	4
Total	211	9 (4.3)	0	13 (6.2)	113	32	45	17

Values in parenthesis represent mortality %

*HOCM* hypertrophic obstructive cardiomyopathy,* DCM* dilated cardiomyopathy,* AVR* aortic valve replacement,* MVR* mitral valve replacement,* MVP* mitral alve repair, *CABG* coronary artery bypass grafting

**Table 18:** (7) Other open-heart operation (total; 820)

	Cases	30-day mortality			Hospital mortality
		Hospital		After discharge	
Total	820	36 (4.4)		0	42 (5.1)

Values in parenthesis represent mortality %

**Table 19:** **Table 5** Thoracic aortic aneurysm (total; 17,498) (1) Dissection (total; 7733)

Replaced site	Stanford type																								
	Acute								Chronic								Concomitant operation					Redo			
	A				B				A				B												
	Cases	30-day mortality		Hospital mortality	Cases	30-day mortality		Hospital mortality	Cases	30-day mortality		Hospital mortality	Cases	30-day mortality		Hospital mortality	AVP	AVR	MVP	MVR	CABG	Cases	30-day mortality		Hospital mortality
		Hospital	After discharge			Hospital	After discharge			Hospital	After discharge			Hospital	After discharge								Hospital	After discharge	
1. Ascending Ao.	2787	220 (7.9)	1 (0.04)	267 (9.6)	1	0	0	0	234	7 (3.0)	0	13 (5.6)	7	2 (28.6)	0	2 (28.6)	182	143	5	7	137	71	10 (14.1)	0	14 (19.7)
2. Aortic root	197	42 (21.3)	0	48 (24.4)	1	0	0	0	60	5 (8.3)	0	8 (13.3)	1	0	0	0	39	181	3	2	52	34	6 (17.6)	0	8 (23.5)
3. Ascending Ao. + Arch	1525	129 (8.5)	0	156 (10.2)	41	5 (12.2)	0	8 (19.5)	295	3 (1.0)	0	10 (3.4)	109	2 (1.8)	0	5 (4.6)	104	52	10	2	75	76	5 (6.6)	0	8 (10.5)
4. Arch + descending Ao.	57	2 (3.5)	0	5 (8.8)	16	5 (31.3)	0	6 (37.5)	24	1 (4.2)	0	2 (8.3)	62	5 (8.1)	0	7 (11.3)	0	0	0	0	5	19	1 (5.3)	0	2 (10.5)
5. Aortic root + Asc. Ao. + Arch	129	21 (16.3)	0	23 (17.8)	0	0	0	0	29	3 (10.3)	0	8 (27.6)	5	0	0	0	24	109	1	1	28	17	1 (5.9)	0	1 (5.9)
6. Descending Ao.	16	1 (6.3)	0	1 (6.3)	41	4 (9.8)	0	7 (17.1)	63	2 (3.2)	0	3 (4.8)	208	11 (5.3)	0	14 (6.7)	0	1	0	0	1	24	4 (16.7)	0	6 (25.0)
7. Thoracoabdominal Ao.	2	0	0	1 (50.0)	11	3 (27.3)	0	4 (36.4)	27	2 (7.4)	0	3 (11.1)	138	7 (5.1)	0	12 (8.7)	0	0	0	0	1	31	4 (12.9)	0	5 (16.1)
8. Extra-anatomical bypass	7	1 (14.3)	0	1 (14.3)	8	0	0	0	3	0	0	0	4	0	0	0	0	0	0	0	0	0	0	0	0
9. Stent graft*^a^	233	18 (7.7)	0	25 (10.7)	277	11 (4.0)	0	16 (5.8)	232	4 (1.7)	0	8 (3.4)	883	18 (2.0)	1 (0.1)	26 (2.9)	8	5	3	0	7	94	4 (4.3)	0	4 (4.3)
1) TEVARl^*b^	105	8 (7.6)	0	11 (10.5)	272	11 (4.0)	0	15 (5.5)	170	1 (0.6)	0	3 (1.8)	835	16 (1.9)	1 (0.1)	24 (2.9)	0	0	0	0	0	77	2 (2.6)	0	2 (2.6)
2) Open stent	128	10 (7.8)	0	14 (10.9)	5	0	0	1 (20.0)	62	3 (4.8)	0	5 (8.1)	48	2 (4.2)	0	2 (4.2)	8	5	3	0	7	17	2 (11.8)	0	2 (11.8)
a) With total arch^*c^	127	10 (7.9)	0	14 (11.0)	4	0	0	1 (25.0)	54	3 (5.6)	0	5 (9.3)	43	2 (4.7)	0	2 (4.7)	8	5	3	0	7	16	2 (12.5)	0	2 (12.5)
b) Without total arch^*d^	1	0	0	0	1	0	0	0	8	0	0	0	5	0	0	0	0	0	0	0	0	1	0	0	0
3) Unspecified	0	0	0	0	0	0	0	0	0	0	0	0	0	0	0	0	0	0	0	0	0	0	0	0	0
Total	4953	434 (8.8)	1 (0.02)	527 (10.6)	396	28 (7.1)	0	41 (10.4)	967	27 (2.8)	0	55 (5.7)	1,417	45 (3.2)	1 (0.1)	66 (4.7)	357	491	22	12	306	366	35 (9.6)	0	48 (13.1)

Values in parenthesis represent mortality %

*Ao* aorta, *AVP* aortic valve repair, *AVR* aortic valve replacement, *MVP* mitral valve repair, *MVR* mitral valve replacement, *CABG* coronary artery bypass grafting, *TEVAR* thoracic endovascular aortic (aneurysm) repair

Acute, within 2 weeks from the onset

*a = *b + *c + *d + unspecified

**Table 20:** (2) Non-dissection (total; 9765)

Replaced site	Unruptured				Ruptured				Concomitant operation					Redo				CPB (−)			
	Cases	30-day mortality		Hospital mortality	Cases	30-day mortality		Hospital mortality	AVP	AVR	MVP	MVR	CABG	Cases	30-day mortality		Hospital mortality	Cases	30-day mortality		Hospital mortality
		Hospital	After discharge			Hospital	After discharge								Hospital	After discharge			Hospital	After discharge	
1. Ascending Ao.	1369	24 (1.8)	0	38 (2.8)	36	4 (11.1)	0	7 (19.4)	82	872	67	50	171	122	5 (4.1)	0	9 (7.4)	–	–	–	–
2. Aortic root	1022	27 (2.6)	0	32 (3.1)	35	8 (22.9)	0	10 (28.6)	250	698	71	19	121	129	17 (13.2)	0	22 (17.1)	–	–	–	–
3. Ascending Ao. + Arch	2139	43 (2.0)	0	75 (3.5)	162	29 (17.9)	4 (2.5)	38 (23.5)	44	181	21	8	351	90	5 (5.6)	0	6 (6.7)	–	–	–	–
4. Arch + descending Ao.	137	10 (7.3)	0	14 (10.2)	22	2 (9.1)	0	4 (18.2)	0	11	0	0	9	7	1 (14.3)	0	2 (28.6)	–	–	–	–
5. Aortic root + Asc. Ao. + Arch	120	2 (1.7)	0	3 (2.5)	2	0	0	0	26	90	3	1	12	10	0	0	1 (10.0)	–	–	–	–
6. Descending Ao.	255	8 (3.1)	0	12 (4.7)	64	11 (17.2)	0	17 (26.6)	0	0	0	0	5	16	4 (25.0)	0	6 (37.5)	8	1 (12.5)	0	1 (12.5)
7. Thoracoabdominal Ao.	390	21 (5.4)	0	28 (7.2)	65	14 (21.5)	0	20 (30.8)	0	0	0	0	0	24	3 (12.5)	0	4 (16.7)	9	0	0	0
8. Extra-anatomical bypass	25	0	1 (4.0)	0	0	0	0	0	0	1	0	0	2	3	0	0	1 (33.3)	10	0	0	1 (10.0)
9. Stent graft^*a^	3528	55 (1.6)	3 (0.1)	95 (2.7)	394	46 (11.7)	0	69 (17.5)	12	14	2	1	50	159	11 (6.9)	0	25 (15.7)	1100	23 (2.1)	1 (0.1)	35 (3.2)
1) TEVAR^*b^	3158	43 (1.4)	3 (0.1)	75 (2.4)	363	42 (11.6)	0	62 (17.1)	6	1	1	0	11	148	8 (5.4)	0	22 (14.9)	1100	23 (2.1)	1 (0.1)	35 (3.2)
2) Open stent	370	12 (3.2)	0	20 (5.4)	31	4 (12.9)	0	7 (22.6)	6	13	1	1	39	11	3 (27.3)	0	3 (27.3)	–	–	–	–
a) With total arch^*c^	285	8 (2.8)	0	16 (5.6)	23	1 (4.3)	0	4 (17.4)	6	13	1	1	35	8	2 (25.0)	0	2 (25.0)	–	–	–	–
b) Without total arch^*d^	85	4 (4.7)	0	4 (4.7)	8	3 (37.5)	0	3 (37.5)	0	0	0	0	4	3	1 (33.3)	0	1 (33.3)	–	–	–	–
3) Unspecified	0	0	0	0	0	0	0	0	0	0	0	0	0	0	0	0	0	0	0	0	0
Total	8985	190 (2.1)	4 (0.04)	297 (3.3)	780	114 (14.6)	4 (0.5)	165 (21.2)	414	1867	164	79	721	560	46 (8.2)	0	76 (13.6)	1,127	24 (2.1)	1 (0.1)	37 (3.3)

Values in parenthesis represent mortality %

*Ao* aorta, *AVP* aortic valve repair, *AVR* aortic valve replacement, *MVP* mitral valve repair, *MVR* mitral valve replacement, *CABG* coronary artery bypass grafting, *TEVAR* thoracic endovascular aortic (aneurysm) repair

*a = *b + *c + *d + unspecified

**Table 21:** **Table 6** Pulmonary thromboembolism (total; 171)

	Cases	30-day mortality		Hospital mortality
		Hospital	After discharge	
Acute	110	15 (13.6)	6 (5.5)	19 (17.3)
Chronic	61	6 (9.8)	0	6 (9.8)
Total	171	21 (12.3)	6 (3.5)	25 (14.6)

Values in parenthesis represent mortality %

**Table 22:** **Table 7** Assisted circulation (total; 1679)

Sites	VAD									Heart–lung assist					
	Device			Results						Method		Results			
	Centrifugal	VAS (extra)	VAS (implant)	Not weaned			Weaned			PCPS	Others	Not weaned		Weaned	
				On going	Death	Transplant	Alive	Deaths	Transplant			Deaths	Transplant	Deaths	Alive
Post cardiotomy															
Left	23	5	5	5	13 (39.4)	0	12	3 (9.1)	0						
Right	2	0	0	0	1 (50.0)	0	1	0	0						
Biventricle															
Right	8	0	0	0	6 (75.0)	0	2	1 (12.5)	0	432	78	259 (50.8)	0	79 (15.5)	157
Left	7	3	0												
Congestive heart failure															
Left	52	41	99	101	56 (29.2)	6	18	7 (3.6)	1						
Right	6	1	0	0	2 (28.6)	0	3	2 (28.6)	0						
Biventricle															
Right	24	6	0	3	16 (53.3)	0	8	2 (6.7)	1	676	61	332 (45.0)	1	111 (15.1)	281
Left	10	16	4												
Respiratory failure										80	40	35 (29.2)	0	15 (12.5)	70
Total	132	72	108	109	94 (30.1)	6	44	15 (4.8)	2	1188	179	626 (45.8)	1	205 (15.0)	508

Values in parenthesis represent mortality %

*VAD* ventricular assist devise,* VAS* ventricular assist system, extra Extracorporeal VAS, implant Implantable VAS, * PCPS* percutaneous cardiopulmonary support

**Table 23:** **Table 8** Heart transplantation (total; 30)

	Cases	30-day mortality		Hospital mortality
		Hospital	After discharge	
Heart transplantation	30	1 (3.3)	0	2 (6.7)
Heart and lung transplantation	0	0	0	0
Total	30	1 (3.3)	0	2 (6.7)

Values in parenthesis represent mortality %

**Table 24:** **Table 9** Pacemaker + ICD (total; 4923)

	Pacemaker			ICD	
	V	A-V	CRT	CRTD	ICD
Initial	570	1,971	94	245	383
Exchange	454	807	29	116	254
Unclear	0	0	0	0	0
Total	1024	2778	123	361	637

*ICD* implantable cardioverter-defibrillator, *CRTD* cardiac resynchronization therapy devise with incorporated ICD devise

In 2014, 6894 open-heart operations for congenital heart disease were performed with overall hospital mortality of 2.3 %. The number of operations for congenital heart disease was quite steady throughout these 10 years (maximum 7,386 cases in 2006), while overall hospital mortality decreased gradually from that of 3.9 % in 2004. In detail, the most common disease was atrial septal defect (1,248 cases); however, its number deceased to 64.3 % of that in 2004, which might be partially due to the recent development of catheter closure of atrial septal defect in Japan. In the last 10 years, hospital mortality for complex congenital heart disease improved in some anomalies such as, complete atrioventricular septal defect (5.4–1.7 %), tetralogy of Fallot (2.5–1.1 %), transposition of the great arteries with and without ventricular septal defect (9.8–3.9 and 7.1–6.6 %, respectively), single ventricle (8.5–4.3 %), and hypoplastic left heart syndrome (27.7–9.8 %). Right heart bypass surgery is now commonly performed (351 bidirectional Glenn procedures excluding 56 Damus–Kaye–Stansel procedures and 397 Fontan-type procedures including total cavopulmonary connection) with acceptable hospital mortality (1.2 and 1.0 %). Norwood type I procedure was performed in 125 cases with relatively low hospital mortality rate of 15.2 %.

As previously mentioned, the number of operations for valvular heart disease increased by 73.8 % in the last 10 years, and the hospital mortality associated with primary single valve replacement was 2.4 and 5.9 % for the aortic and the mitral position, while that for primary mitral valve repair was 1.1 %. However, hospital mortality rate for redo valve surgery was still high and was 9.4 and 7.8 % for aortic and mitral procedure, respectively. Finally, overall hospital mortality did not show dramatic improvement during the last 10 years (3.8 % in 2004 and 3.1 % in 2014), which might be partially due to the recent progression of age of the patients. Repair of the valve became popular procedure (397 cases in the aortic, 6527 cases in the mitral, and 5066 cases in the tricuspid), and mitral valve repair constituted 29.8 % of all valvular heart disease operation and 59.6 % of all mitral valve procedure (10,957 procedures), which are similar to those of the last 5 years and increased compared with those of 2004 (23.6 and 42.8 %, respectively). Aortic and mitral valve replacements with bioprosthesis were performed in 10,220 cases and 2,765 cases, respectively, with the number consistently increasing in the aortic position. The ratio of prostheses changed dramatically during the last 10 years and the usage of bioprosthesis is 77.5 % at the aortic position (36.7 % in 2004) and 25.2 % at the mitral position (14.8 % in 2004). CABG as a concomitant procedure performed in 17.3 % of operations for all valvular heart disease (13.3 % in 2004).

Isolated CABG was performed in 14,454 cases which were only 72.5 % of that of 10 years ago (2004). Among these 14,454 cases, off-pump CABG was intended in 9,006 cases (62.3 %) with a success rate of 98.3 %, so final success rate of off-pump CABG was 61.2 %. The percentage of intended off-pump CABG reached 60.3 % in 2004, and then was kept over 60 % until now. In 14,454 isolated CABG patients, 95.4 % of them received at least one arterial graft, while all arterial graft CABG was performed only 21.4 % of them.

The operative and hospital mortality rates associated with primary elective CABG procedures in 12335 cases were 0.8 and 1.3 %, respectively. Similar data analysis of CABG, including primary/redo and elective/emergency data, was begun in 2003, and the operative and hospital mortality rates associated with primary elective CABG procedures in 2003 were 1.0 and 1.5 %, respectively, so operative results of primary CABG has been stable, while hospital mortality of primary emergency CABG in 1,959 cases was still high and was 7.9 %. During these 10 years, the results of conversion from off-pump CABG improved both in conversion rate (3.1–1.7 %) and in hospital mortality (10.4–4.5 %).

A total of 1175 patients underwent surgery for complications of myocardial infarction, including 329 operations for a left ventricular aneurysm or ventricular septal perforation or cardiac rupture and 261 operations for ischemic mitral regurgitation.

Operations for arrhythmia were performed mainly as a concomitant procedure in 3855 cases with satisfactory mortality (1.6 % hospital mortality) including 3,486 MAZE procedures. MAZE procedure has become quite popular procedure when compared with that in 2004 (1837 cases).

Operations for thoracic aortic dissection were performed in 7733 cases. For 4953 Stanford type A acute aortic dissections, hospital mortality remained high and was 10.6 %. Operations for a non-dissected thoracic aneurysm were carried out in 9765 cases, with overall hospital mortality of 4.7 %. The hospital mortality associated with unruptured aneurysm was 3.3 %, and that of ruptured aneurysm was 21.2 %, which remains markedly high.

The number of stent graft procedures remarkably increased recently. A total of 1,625 patients with aortic dissection underwent stent graft placement: thoracic endovascular aortic repair (TEVAR) in 1,382 cases and open stent grafting in 243 cases. The number of TEVAR for type B chronic aortic dissections increased from 69 cases in 2004 to 835 cases in 2014. The hospital mortality rates associated with TEVAR for type B aortic dissection were 5.5 % in acute cases and 2.9 % for chronic cases, respectively.

A total of 3922 patients with non-dissected aortic aneurysm underwent stent graft placement; TEVAR in 3521 cases (12.4 % increase compared with that in 2013) and open stent grafting in 401 cases (145 % increase compared with that in 2013). The reason of dramatic increase in open stent grafting might be due to commercially availability since 2014. The hospital mortality rates for TEVAR were 2.4 and 17.1 % for non-ruptured and ruptured aneurysm, respectively.

In summary, the total cardiovascular operations increased during 2014 by 1141 cases with steadily improving results in almost all categories throughout these 10 years.

### (B) General thoracic surgery

**Fig. 2:**
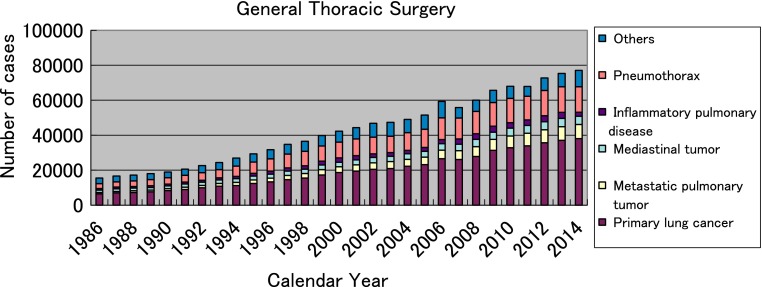
General thoracic surgery

The total number of operations reported in 2014 in general thoracic surgery has reached 77070, which means 1.74-fold of that in 2001, and increased by 1764 cases compared with that in 2013 (Fig. 2, Table 10).

**Table 25:** **Table 10** Total entry cases of general thoracic surgery during 2014

	Cases	%
Benign pulmonary tumor	2171	2.8
Primary lung cancer	38,085	49.4
Other primary malignant pulmonary tumor	359	0.5
Metastatic pulmonary tumor	8057	10.5
Tracheal tumor	118	0.2
Mesothelioma	673	0.9
Chest wall tumor	698	0.9
Mediastinal tumor	4685	6.1
Thymectomy for MG without thymoma	188	0.2
Inflammatory pulmonary disease	2287	3.0
Empyema	2608	3.4
Bullous disease excluding pneumothorax	415	0.5
Pneumothorax	14,572	18.9
Chest wall deformity	217	0.3
Diaphragmatic hernia including traumatic	55	0.1
Chest trauma excluding diaphragmatic hernia	394	0.5
Lung transplantation	60	0.1
Others	1428	1.9
Total	77,070	100.0

The number of operations for primary lung cancer was 38085 in 2014 (Table 10), showing the steady increase (31,301; 2009, 32,801; 2010, 33,878; 2011, 35,667; 2012, 37,008; 2013), and 1.95-fold of the number of operations in 2001. Surgery for lung cancer consists 49.4 % of all the general thoracic surgery.

Surgery for benign pulmonary tumor was 2171 in 2014 (Table 11).

**Table 26:** **Table 11** 1. Benign pulmonary tumor

	Cases	30-day mortality		Hospital mortality
		Hospital	After discharge	
Hamartoma	481	0	0	0
Sclerosing hemangioma	103	0	0	0
Papilloma	18	0	0	0
Mucous gland adenoma bronchial	7	0	0	0
Fibroma	129	0	0	0
Lipoma	6	0	0	0
Neurogenic tumor	17	0	0	0
Clear cell tumor	2	0	0	0
Leiomyoma	19	0	0	0
Chondroma	5	0	0	0
Inflammatory myofibroblastic tumor	1	0	0	0
Pseudolymphoma	32	0	0	0
Histiocytosis	23	0	0	0
Teratoma	0	0	0	0
Others	1328	2 (0.2)	1 (0.1)	6 (0.5)
Total	2171	2 (0.1)	1 (0.05)	6 (0.3)

Values in parenthesis represent mortality %

Further information of primary malignant pulmonary tumors is shown in Tables 12 and 13. Among lung cancer subtypes, adenocarcinoma comprises an overwhelming percentage of 69.2 % of the total lung cancer surgery, followed by squamous cell carcinoma of 19.3 %. Limited resection by wedge resection or segmentectomy was performed in 9581 lung cancer patients, which is 25.2 % of the entire cases. Lobectomy was performed in 27,584 patients, which is 72.4 % of the entire cases. Sleeve lobectomy was done in 471 patients. Pneumonectomy was done in 521 patients which is 1.4 % of the entire cases.

**Table 27:** **Table 12** 2. Primary malignant pulmonary tumor

	Cases	30-day mortality		Hospital mortality
		Hospital	After discharge	
2. Primary malignant pulmonary tumor	38,444	104 (0.3)	59 (0.2)	269 (0.7)
Lung cancer	38,085	103 (0.3)	59 (0.2)	266 (0.7)
Adenocarcinoma	26,338	33 (0.1)	23 (0.1)	82 (0.3)
Squamous cell carcinoma	7367	46 (0.6)	22 (0.3)	127 (1.7)
Large cell carcinoma	835	5 (0.6)	6 (0.7)	10 (1.2)
(*LCNEC*)	*462*	*4* (0.9)	*1* (0.2)	*8* (1.7)
Small cell carcinoma	601	1 (0.2)	1 (0.2)	9 (1.5)
Adenosquamous carcinoma	548	7 (1.3)	0	14 (2.6)
Carcinoma with pleomorphic, sarcomatoid or sarcomatous elements	528	6 (1.1)	2 (0.4)	12 (2.3)
Carcinoid	198	0	0	0
Carcinomas of salivary-gland type	45	0	0	0
Unclassified	55	2 (3.6)	0	4 (7.3)
Multiple lung cancer	1227	1 (0.1)	3 (0.2)	6 (0.5)
Others	343	2 (0.6)	2 (0.6)	2 (0.6)
Wedge resection	5438	4 (0.1)	4 (0.1)	20 (0.4)
Segmental excision	4143	2 (0.05)	3 (0.1)	13 (0.3)
(*Sleeve segmental excision*)	*16*	*0*	*0*	*0*
Lobectomy	27,584	82 (0.3)	51 (0.2)	198 (0.7)
(*Sleeve lobectomy*)	*471*	*5* (1.1)	*7* (1.5)	*10* (2.1)
Pneumonectomy	521	8 (1.5)	0	20 (3.8)
(*Sleeve pneumonectomy*)	*13*	*0*	*0*	*1* (7.7)
Other bronchoplasty	46	2 (4.3)	0	2 (4.3)
Pleuropneumonectomy	1	0	0	0
Others	343	5 (1.5)	1 (0.3)	10 (2.9)
Sarcoma	40	0	0	0
AAH	126	0	0	0
Others	193	1 (0.5)	0	3 (1.6)

Values in parenthesis represent mortality %

**Table 28:** **Table 13** Details of lung cancer operation

	Cases
c-Stage (TNM)	
Ia	22,809
Ib	7213
IIa	2982
IIb	1780
IIIa	2505
IIIb	204
IV	481
NA	111
Total	38,085
Sex	
Male	23,540
Female	14,516
NA	29
Total	38,085
Cause of death	
Cardiovascular	23
Pneumonia	47
Pyothorax	4
Bronchopleural fistula	16
Respiratory failure	41
Pulmonary embolism	11
Interstitial pneumonia	78
Brain infarction or bleeding	14
Others	80
Unknown	11
Total	325
p-Stage	
0 (pCR)	295
Ia	19,666
Ib	7601
IIa	3213
IIb	2087
IIIa	3761
IIIb	179
IV	1072
NA	211
Total	38,085
Age	
<20	85
20–29	33
30–39	219
40–49	1009
50–59	3646
60–69	12,731
70–79	15,765
80–89	4532
≥90	58
NA	7
Total	38,085

There were 103 patients who died without discharge within 30 days after lung cancer surgery, and 59 patients who were discharged from hospital but died within 30 days after lung cancer surgery, indicating that 162 patients died within 30 days after lung cancer surgery (30-day mortality rate; 0.42 %). There were 266 patients died without discharge (hospital mortality rate; 0.70 %). 30-day mortality rate in regard to procedures is 0.12 % in segmentectomy, 0.48 % in lobectomy, and 1.53 % in pneumonectomy. Interstitial pneumonia was the leading cause of death after lung cancer surgery, followed by pneumonia, respiratory failure, cardiovascular event, and bronchopleural fistula.

Surgery for metastatic pulmonary tumors is denoted in Table 14. The number of patients undergoing operations for metastatic pulmonary tumor was 8057 in 2014 with steady increase similarly to lung cancer surgery (6248; 2009, 6748: 2010, 7210; 2011, 7403; 2012, 7829; 2013). Colorectal cancer was by far the leading primary malignancy indicated for resection of metastatic tumors, which comprises 48.4 % of the entire cases.

**Table 29:** **Table 14** 3. Metastatic pulmonary tumor

	Cases	30-day mortality		Hospital mortality
		Hospital	After discharge	
3. Metastatic pulmonary tumor	8057	17 (0.2)	8 (0.1)	30 (0.4)
Colo-rectal	3902	2 (0.1)	0	5 (0.1)
Hepatobiliary/pancreatic	388	2 (0.5)	0	2 (0.5)
Uterine	387	0	0	0
Mammary	445	0	0	0
Ovarian	56	0	0	0
Testicular	84	0	0	0
Renal	618	3 (0.5)	2 (0.3)	3 (0.5)
Skeletal	148	0	1 (0.7)	0
Soft tissue	235	0	1 (0.4)	2 (0.9)
Otorhinolaryngological	422	2 (0.5)	1 (0.2)	2 (0.5)
Pulmonary	497	8 (1.6)	1 (0.2)	11 (2.2)
Others	875	0	2 (0.2)	5 (0.6)

Values in parenthesis represent mortality %

118 tracheal tumors were operated in 2014 (Table 15). Squamous cell carcinoma and adenoid cystic carcinoma were frequent primary tracheal tumor.

**Table 30:** **Table 15** 4. Tracheal tumor

	Cases	30-day mortality		Hospital mortality
		Hospital	After discharge	
4. Tracheal tumor	118	4 (3.4)	1 (0.8)	10 (8.5)
(A) Primary malignant tumor (histological classification)				
Squamous cell carcinoma	15	0	0	1 (6.7)
Adenoid cystic carcinoma	9	0	0	0
Mucoepidermoid carcinoma	2	0	0	0
Others	10	0	0	0
Total	36	0	0	1 (2.8)
(B) Metastatic/invasive malignant tumor, e.g. invasion of thyroid cancer	48	4 (8.3)	1 (2.1)	9 (18.8)
(C) Benign tracheal tumor (histological classification)				
Papilloma	0	0	0	0
Adenoma	3	0	0	0
Neurofibroma	1	0	0	0
Chondroma	0	0	0	0
Leiomyoma	3	0	0	0
Others	27	0	0	0
Histology unknown	0	0	0	0
Total	34	0	0	0
Operation				
Sleeve resection with reconstruction	13	0	0	1 (7.7)
Wedge with simple closure	0	0	0	0
Wedge with patch closure	0	0	0	0
Total laryngectomy with tracheostomy	0	0	0	0
Others	29	0	0	0
Unknown	0	0	0	0
Total	42	0	0	1 (2.4)

Values in parenthesis represent mortality %

673 tumors of the pleural origin were operated in 2014 (Table 16). Diffuse malignant pleural mesothelioma was the most frequent histology. Total pleurectomy was performed in 73 patents and surpassed extrapleural pneumonectomy which was the most frequently chosen operative method in 2013. Hospital mortality rate was 4.1 % after total pleurectomy and 4.3 % after extrapleural pneumonectomy in 2014.

**Table 31:** **Table 16** 5. Tumor of pleural origin

	Cases	30-day mortality		Hospital mortality
		Hospital	After discharge	
Histological classification				
Solitary fibrous tumor	122	0	0	0
Diffuse malignant pleural mesothelioma	283	3 (1.1)	0	10 (3.5)
Localized malignant pleural mesothelioma	26	0	0	1 (3.8)
Others	242	3 (1.2)	2 (0.8)	9 (3.7)
Total	673	6 (0.9)	2 (0.3)	20 (3.0)
Operative procedure				
Extrapleural pneumonectomy	70	1 (1.4)	0	3 (4.3)
Total pleurectomy	73	1 (1.4)	0	3 (4.1)
Others	140	1 (0.7)	0	4 (2.9)
Total	283	3 (1.1)	0	10 (3.5)

Values in parenthesis represent mortality %

698 chest wall tumors were resected in 2014 (Table 17). 362 cases (51.9 %) were benign. Among 336 malignant chest wall tumors, 208 cases (61.9 %) were metastatic tumors.

**Table 32:** **Table 17** 6. Chest wall tumor

	Cases	30-day mortality		Hospital mortality
		Hospital	After discharge	
Primary malignant tumor	128	1 (0.8)	0	5 (3.9)
Metastatic malignant tumor	208	0	1 (0.5)	3 (1.4)
Benign tumor	362	0	0	0
Total	698	1 (0.1)	1 (0.1)	8 (1.1)

Values in parenthesis represent mortality %

Table 18 denotes surgery for mediastinal tumors. 4685 mediastinal tumors were operated in 2014. There were 2104 thymic epithelial tumors (1773 thymomas, 296 thymic carcinomas, and 35 thymic neuroendocrine carcinoma including carcinoid), followed by 932 congenital cysts, 481 neurogenic tumors, 214 lymphatic tumors, and 122 germ cell tumors.

**Table 33:** **Table 18** 7. Mediastinal tumor

	Cases	30-day mortality		Hospital mortality
		Hospital	After discharge	
7. Mediastinal tumor	4685	5 (0.1)	2 (0.04)	17 (0.4)
Thymoma*	1773	5 (0.3)	0	9 (0.5)
Thymic cancer	296	0	0	1 (0.3)
Thymus carcinoid	35	0	0	0
Germ cell tumor	122	0	0	0
*Benign*	*87*	*0*	*0*	*0*
*Malignant*	*35*	*0*	*0*	*0*
Neurogenic tumor	481	0	0	0
Congenital cyst	932	0	1 (0.1)	5 (0.5)
Goiter	75	0	0	1 (1.3)
Lymphatic tumor	214	0	0	0
Excision of pleural recurrence of thymoma	43	0	0	0
Thymolipoma	14	0	0	0
Others	700	0	1 (0.1)	1 (0.1)

Values in parenthesis represent mortality %

* Includes those with myasthenia gravis

Thymectomy for myasthenia gravis was done in 495 patients (Table 19). Among them, 307 patients were associated with thymoma, and the remaining 188 patients were not associated with thymoma.

**Table 34:** **Table 19** 8. Thymectomy for myasthenia gravis

	Cases	30-day mortality		Hospital mortality
		Hospital	After discharge	
8. Thymectomy for myasthenia gravis	495	1 (0.2)	0	1 (0.2)
With thymoma	307	1 (0.3)	0	1 (0.3)

Values in parenthesis represent mortality %

Lung resection for inflammatory lung diseases were done in 2287 patients in 2014 (Table 20). Inflammatory pseudotumor comprised 24.7 % of the entire cases, followed by atypical mycobacterium infection (21.9 %) and fungal infections (15.1 %).

**Table 35:** **Table 20** 9. Operation for non-neoplastic disease (A) Inflammatory pulmonary disease

	Cases	30-day mortality		Hospital mortality
		Hospital	After discharge	
9. Operation for non-neoplastic disease	21,976	197 (0.9)	14 (0.1)	425 (1.9)
(A) Inflammatory pulmonary disease	2287	6 (0.3)	2 (0.1)	17 (0.7)
Tuberculous infection	73	0	0	0
Mycobacterial infection	501	1 (0.2)	1 (0.2)	3 (0.6)
Fungal infection	345	1 (0.3)	1 (0.3)	6 (1.7)
Bronchiectasis	67	0	0	1 (1.5)
Tuberculous nodule	133	0	0	0
Inflammatory pseudo tumor	566	0	0	0
Interpulmonary lymph node	63	0	0	0
Others	539	4 (0.7)	0	7 (1.3)

Values in parenthesis represent mortality %

2,608 operations for empyema were reported in 2014 (Table 21). There were 1911 patients (73.3 %) with acute empyema and 698 patients with chronic empyema. Bronchopleural fistula was associated in 469 patients (24.5 %) with acute empyema and 345 patients (49.5 %) with chronic empyema. It should be noted that hospital mortality was as high as 15.1 % in patients of acute empyema with fistula.

**Table 36:** **Table 21** 9. Operation for non-neoplastic disease (B) Empyema

	Cases	30-day mortality		Hospital mortality
		Hospital	After discharge	
Acute empyema	1911	52 (2.7)	3 (0.2)	126 (6.6)
With fistula	469	28 (6.0)	1 (0.2)	71 (15.1)
Without fistula	1425	23 (1.6)	2 (0.1)	52 (3.6)
Unknown	17	1 (5.9)	0	3 (17.6)
Chronic empyema	697	14 (2.0)	1 (0.1)	38 (5.5)
With fistula	345	12 (3.5)	1 (0.3)	27 (7.8)
Without fistula	328	2 (0.6)	0	10 (3.0)
Unknown	24	0	0	1 (4.2)
Total	2608	66 (2.5)	4 (0.2)	164 (6.3)

Values in parenthesis represent mortality %

Operation for descending necrotizing mediastinitis was done in 103 patients in 2014 (Table 22). Hospital mortality rate was 8.7 %.

Operation for bullous diseases was done in 415 patients in 2014 (Table 23). Lung volume reduction surgery was done in only 28 patients, while emphysematous bulla was the principal target of operation.

**Table 37:** **Table 22** 9. Operation for non-neoplastic disease (C) Descending necrotizing mediastinitis

	Cases	30-day mortality		Hospital mortality
		Hospital	After discharge	
(C) Descending necrotizing mediastinitis	103	6 (5.8)	0	9 (8.7)

Values in parenthesis represent mortality %

**Table 38:** **Table 23** 9. Operation for non-neoplastic disease (D) Bullous disease

	Cases	30-day mortality		Hospital mortality
		Hospital	After discharge	
(D) Bullous disease	415	1 (0.2)	0	1 (0.2)
Emphysematous bulla	322	1 (0.3)	0	1 (0.3)
Bronchogenic cyst	18	0	0	0
Emphysema with volume reduction surgery	28	0	0	0
Others	47	0	0	0

Values in parenthesis represent mortality %

*LVRS* lung volume reduction surgery

14,572 operations for pneumothorax were reported in 2014 (Table 24).

**Table 39:** **Table 24** 9. Operation for non-neoplastic disease (E) Pneumothorax

	Cases	30-day mortality		Hospital mortality
		Hospital	After discharge	
(E) Pneumothorax	14,572	60 (0.4)	8 (0.1)	133 (0.9)
*Spontaneous pneumothorax*				
Operative procedure				
Bullectomy	3410	3 (0.1)	0	12 (0.4)
Bullectomy with additional procedure	7625	2 (0.03)	1 (0.01)	7 (0.1)
Coverage with artificial material	7241	2 (0.03)	0	6 (0.1)
Parietal pleurectomy	51	0	0	1 (2.0)
Coverage and parietal pleurectomy	92	0	0	0
Others	241	0	1 (0.4)	0
Others	905	8 (0.9)	0	12 (1.3)
Unknown	8	0	0	0
Total	11,948	13 (0.1)	1 (0.01)	31 (0.3)
*Secondary pneumothorax*				
Associated disease				
COPD	1763	18 (1.0)	2 (0.1)	51 (2.9)
Tumorous disease	84	7 (8.3)	3 (3.6)	14 (16.7)
Catamenial	148	0	0	0
LAM	47	0	0	0
Others (excluding pneumothorax by trauma)	582	22 (3.8)	2 (0.3)	37 (6.4)
Unknown				
Operative procedure				
Bullectomy	372	2 (0.5)	1 (0.3)	3 (0.8)
Bullectomy with additional procedure	1509	16 (1.1)	2 (0.1)	37 (2.5)
Coverage with artificial material	1423	16 (1.1)	2 (0.1)	37 (2.6)
Parietal pleurectomy	9	0	0	0
Coverage and parietal pleurectomy	18	0	0	0
Others	59	0	0	0
Others	735	29 (3.9)	4 (0.5)	62 (8.4)
Unknown	8	0	0	0
Total	2624	47 (1.8)	7 (0.3)	102 (3.9)

Values in parenthesis represent mortality %

The number of operations for spontaneous pneumothorax was 11,948. Among them, 3410 patients (28.5 %) underwent bullectomy alone, while additional procedure was performed in 7625 patients (63.8 %).

The number of operations for secondary pneumothorax was 2624. COPD was by far the most prevalent associated disease (67.2 %). It should be noted that hospital mortality rate of operation for pneumothorax associated with tumorous disease was as high as 16.7 %.

217 cases of surgery for chest wall deformity were reported in 2014 survey (Table 25). This number might be underestimated compared with the real number of operations, because chest wall deformity is more likely to be treated in the institutes which are not associated with JATS.

**Table 40:** **Table 25** 9. Operation for non-neoplastic disease (F) Chest wall deformity

	Cases	30-day mortality		Hospital mortality
		Hospital	After discharge	
(F) Chest wall deformity	217	0	0	0
Funnel chest	209	0	0	0
Others	8	0	0	0

Diaphragmatic hernia was treated by surgery in 55 patients in 2014 (Table 26).

**Table 41:** **Table 26** 9. Operation for non-neoplastic disease (G) Diaphragmatic hernia

	Cases	30-day mortality		Hospital mortality
		Hospital	After discharge	
(G) Diaphragmatic hernia	55	1 (1.8)	0	1 (1.8)
Congenital	22	0	0	0
Traumatic	9	0	0	0
Others	24	1 (4.2)	0	1 (4.2)

Values in parenthesis represent mortality %

Chest trauma was treated by surgery in 394 patients in 2014 (Table 27).

**Table 42:** **Table 27** 9. Operation for non-neoplastic disease (H) Chest trauma

	Cases	30-day mortality		Hospital mortality
		Hospital	After discharge	
(H) Chest trauma	394	29 (7.4)	0	36 (9.1)

Values in parenthesis represent mortality %

Table 28 denotes operations for other diseases, including 77 arteriovenous malformations and 104 pulmonary sequestrations.

**Table 43:** **Table 28** 9. Operation for non-neoplastic disease (I) Other respiratory surgery

	Cases	30-day mortality		Hospital mortality
		Hospital	After discharge	
(I) Other respiratory surgery	1325	28 (2.1)	0	64 (4.8)
Arteriovenous malformation*	77	0	0	0
Pulmonary sequestration	104	0	0	0
Postoperative bleeding air leakage	386	11 (2.8)	0	30 (7.8)
Chylothorax	65	1 (1.5)	0	2 (3.1)
Others	693	16 (2.3)	0	32 (4.6)

Values in parenthesis represent mortality %

Table 29 denotes lung transplantation in 2014. A total of 60 lung transplantations were performed in 2014. The number of patients undergoing lung transplantation from brain-dead donors and living-related donors was 40 and 20, respectively. The number of lung transplantation is almost constant these several years, and lung transplantation is still dependent on living-related donors in Japan.

**Table 44:** **Table 29** 10. Lung transplantation

	Cases	30-day mortality		Hospital mortality
		Hospital	After discharge	
Single lung transplantation from brain dead donor	23	0	0	0
Bilateral lung transplantation from brain dead donor	17	0	0	0
Lung transplantation from living donor	20	0	0	2 (10.0)
Total of lung transplantation	60	0	0	2 (3.3)
Donor of living donor lung transplantation	37	0	0	0

Values in parenthesis represent mortality %

Details of tracheabronchoplasty, pediatric surgery, and combined resection of neighboring organs are denoted in Tables 30, 31, 32, and 33.

**Table 45:** **Table 30** 11. Tracheobronchoplasty

	Cases	30-day mortality		Hospital mortality
		Hospital	After discharge	
11. Tracheobronchoplasty	649	9 (1.4)	7 (1.1)	16 (2.5)
Trachea	27	0	0	1 (3.7)
Sleeve resection with reconstruction	20	0	0	1 (5.0)
Wedge with simple closure	0	0	0	0
Wedge with patch closure	0	0	0	0
Total laryngectomy with tracheostomy	0	0	0	0
Others	7	0	0	0
Carinal reconstruction	28	2 (7.1)	0	2 (7.1)
Sleeve pneumonectomy	15	0	0	1 (6.7)
Sleeve lobectomy	476	5 (1.1)	7 (1.5)	10 (2.1)
Sleeve segmental excision	22	0	0	0
Bronchoplasty without lung resection	13	1 (7.7)	0	1 (7.7)
Others	68	1 (1.5)	0	1 (1.5)

Values in parenthesis represent mortality %

**Table 46:** **Table 31** 12. Pediatric surgery

	Cases	30-day mortality		Hospital mortality
		Hospital	After discharge	
12. Pediatric surgery	580	3 (0.5)	0	7 (1.2)

Values in parenthesis represent mortality %

**Table 47:** **Table 32** 13. Combined resection of neighboring organ(s)

Organ resected	Cases	30-day mortality		Hospital mortality
		Hospital	After discharge	
13. Combined resection of neighboring organ(s)	1408	7 (0.5)	3 (0.2)	25 (1.8)
(A) Primary lung cancer (organ resected)				
Aorta	16	0	0	1 (6.3)
Superior vena cava	26	0	0	2 (7.7)
Brachiocephalic vein	13	1 (7.7)	0	1 (7.7)
Pericardium	143	1 (0.7)	1 (0.7)	4 (2.8)
Pulmonary artery	158	1 (0.6)	0	2 (1.3)
Left atrium	30	0	0	0
Diaphragm	51	0	0	0
Chest wall (including ribs)	360	3 (0.8)	2 (0.6)	17 (4.7)
Vertebra	16	1 (6.3)	0	2 (12.5)
Esophagus	9	0	0	0
Total	822	7 (0.9)	3 (0.4)	29 (3.5)
(B) Mediastinal tumor (organ resected)				
Aorta	2	0	0	1 (50.0)
Superior vena cava	59	0	0	1 (1.7)
Brachiocephalic vein	89	0	0	0
Pericardium	340	2 (0.6)	0	3 (0.9)
Pulmonary artery	3	0	0	0
Left atrium	0	0	0	0
Diaphragm	34	0	0	1 (2.9)
Chest wall (including ribs)	9	0	0	0
Vertebra	13	0	0	0
Esophagus	4	0	0	0
Lung	461	0	0	0
Total	1014	2 (0.2)	0	6 (0.6)

Values in parenthesis represent mortality %

**Table 48:** **Table 33** 14. Operation of lung cancer invading the chest wall of the apex

	Cases	30-day mortality		Hospital mortality
		Hospital	After discharge	
14. Operation of lung cancer invading the chest wall of the apex	737	2 (0.3)	5 (0.7)	15 (2.0)

Values in parenthesis represent mortality %

Includes tumors invading the anterior apical chest wall and posterior apical chest wall (superior sulcus tumor, so-called Pancoast type)

Committee for Scientific Affairs in JATS changed the method of surveying general thoracic surgery in 2014. JATS had investigated the number of diseases and operative procedures based on questionnaires until 2013 surveys, but JATS started to collect the number of procedures in general thoracic surgery using the database in National Clinical Database (NCD) registry. There were some differences in definition in VATS procedure between surveys by JATS before 2013 and that using NCD after 2014. While the length of skin incision in definition of VATS procedure had been less than 8 cm by JATS survey before 2013 following Swanson et al’s proposal [1], NCD registry did not limit the length of skin incision in VATS procedures. On the other hand, NCD required the surgeons to choose the approach among complete VATS procedure without thoracotomy, the procedure using both thoracotomy and VATS which includes hybrid approach, and conventional thoracotomy without VATS procedure. It is presumed that hybrid approach was included in VATS procedure as far as the skin incision was shorter than 8 cm in JATS survey before 2013, but this does not seem to apply to survey in 2014 based on NCD registry, suggesting possible inconsistency in comparison between JATS survey before 2013 and NCD 2014 registry. In this report, therefore, analysis with regard to VATS procedure was not conducted.

### (C) Esophageal surgery

**Fig. 3:**
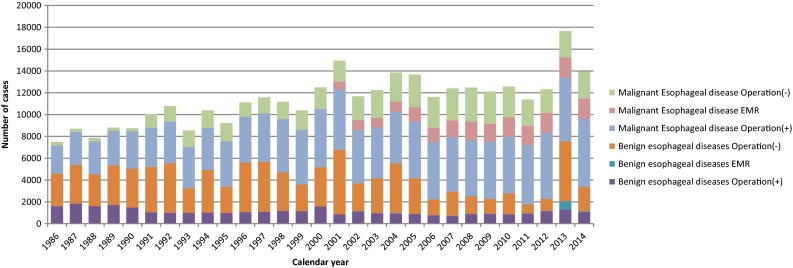
Annual trend of in-patients with esophageal diseases. *EMR* endoscopic mucosal resection (including endoscopic submucosal)

During 2014 alone, a total of 13,958 patients with esophageal diseases were registered from 601 institutions (response rate: 96.0 %) which affiliated to the Japanese Association for Thoracic Surgery and/or to the Japan Esophageal Society. Among these institutions, those where 20 or more patients underwent esophageal surgeries within the year of 2014 were 133 institutions (22.1 %), which shows no definite shift of esophageal operations to high volume institutions when compared to the data of 2013 (33.3 %) (Table 34) Of 3,956 patients with a benign esophageal disease, 1660 (42.0 %) patients underwent surgery, and 57 (1.4 %) patients underwent endoscopic resection, while 2239 (56.6 %) patients did not undergo any surgical treatment. (Table 35) Of 10,638 patients with a malignant esophageal tumor, 8135 (76.5 %) patients underwent resection, esophagectomy for 6247 (59.0 %) and endoscopic mucosal resection (EMR) or endoscopic submucosal dissection (ESD) for 1851 (17.5 %), while 2492 (23.5 %) patients did not undergo any resection. (Tables 36, 37) The patients registered, particularly those undergoing ESD or EMR for a malignant esophageal disease, have been increasing since 1990 (Fig. 3).

**Table 49:** **Table 34** Distribution of number of esophageal operations in 2014 in each institution

Esophageal surgery			
Number of operations in 2014	Benign esophageal diseases	Malignant esophageal disease	Benign + malignant
*0*	289	136	98
1–4	245	148	145
5–9	45	120	117
10–19	17	81	108
20–29	3	36	39
30–39	1	23	27
40–49	0	20	25
≧50	1	37	42
Total	601	601	601

**Table 50:** **Table 35** Benign esophageal diseases

	Operation (+)										Endoscopic resection	Operation (−)	Total
	Number of patients			Hospital mortality									
	Total	Open	T/L*3	Open Surgery			T/L*3			Total			
				~30 days	31–90 days	Total (including after 91 days mortality)	~30 days	31–90 days	Total (including after 91 days mortality)				
1. Achalasia	338	179	159	1 (0.6)	0	1 (0.6)	0	0	0	1 (0.3)		52	390
2. Benign tumor	111	73	38	0	0	0	0	0	0	0	43	18	172
(1) Leiomyoma	70	43	27	0	0	0	0	0	0	0	17	9	96
(2) Cyst	12	7	5	0	0	0	0	0	0	0	0	0	12
(3) Others	29	23	6	0	0	0	0	0	0	0	26	6	61
(4) Not specified	0	0	0	0	0	0	0	0	0	0	0	3	3
3. Diverticulum	55	39	16	0	0	0	0	0	0	0		17	72
4. Hiatal hernia	739	423	316	2 (0.5)	2 (0.5)	4 (0.9)	1 (0.3)	1 (0.3)	2 (0.6)	6 (0.8)		193	932
5. Spontaneous rupture of the esophagus	95	87	8	4 (4.6)	1 (1.1)	5 (5.7)	0	0	0	5 (5.3)		13	108
6. Esophagotracheal fistula	18	17	1	0	0	0	0	0	0	0		12	30
7. Congenital esophageal atresia	51	47	4	0	1 (2.1)	1 (2.1)	0	0	0	1 (2.0)		1	52
8. Congenital esophageal stenosis	10	9	1	0	0	0	0	0	0	0		4	14
9. Corrosive stricture of the esophagus	11	8	3	0	0	0	0	0	0	0		10	21
10. Esophagitis, esophageal ulcer	87	61	26	0	0	0	0	0	0	0		1199	1286
11. Esophageal varices	70	67	3	2 (3.0)	0	2 (3.0)	0	0	0	2 (2.9)		685	755
(1) Laparotomy	9	6	3	0	0	0	0	0	0	0			9
(2) Sclerotherapy												201	201
(3) EVL												344	344
12. Others	75	62	13	5 (8.1)	0	5 (8.1)	0	0	0	5 (6.7)	14	35	124
Total	1660	1072	588	14 (1.3)	4 (0.4)	18 (1.7)	1 (0.2)	1 (0.2)	2 (0.3)	20 (1.2)	57	2239	3956

Values in parenthesis represent mortality %

*T/L* thoracoscopic and/or laparoscopic

**Table 51:** **Table 36** Malignant esophageal diseases (histologic classification)

	Resection (+)	Resection (−)	Total
Carcinomas	8100	2495	10,595
1. Squamous cell carcinoma	7233	2355	9588
2. Basaloid (-squamous) carcinoma	79	2	81
3. Carcinosarcoma	43	3	46
4. Adenocarcinoma in the Barrett’s esophagus	319	21	340
5. Other adenocarcinoma	350	67	417
6. Adenosquamous carcinoma	22	5	27
7. Mucoepidermoid carcinoma	2	0	2
8. Adenoid cystic carcinoma	1	1	2
9. Endcrine cell carcinoma	34	24	58
10. Undifferentiated carcinoma	7	4	11
11. Others	10	13	23
Other malignancies	35	8	43
1. Malignant non-epithelial tumors	8	2	10
2. Malignant melanoma	20	5	25
3. Other malignant tumors	7	1	8
Not specified	0	0	0
Total	8135	2503	10,638

Resection: including endoscopic resection

**Table 52:** **Table 37** Malignant esophageal disease (clinical characteristics)

	Operation (+)				EMR or ESD	Operation (−)	Total
	Cases	Hospital mortality					
		~30 days	31–90 days	Total (including after 91 days mortality)			
1. Esophageal cancer	6247	47 (0.8)	46 (0.7)	128 (2.0)	1851	2492	10,584
Location							
(1) Cervical esophagus	258	0	1 (0.4)	3 (1.2)	76	178	512
(2) Thoracic esophagus	5041	45 (0.9)	39 (0.8)	112 (2.2)	1501	2133	8675
(3) Abdominal esophagus	644	2 (0.3)	3 (0.5)	7 (1.1)	100	117	861
(4) Multiple cancers	301	0	3 (1.0)	6 (2.0)	174	61	536
(5) Others/not described	3	0	0	0	0	3	0
Tumor depth							
(A) Superficial cancer (T1)	1892	9 (0.5)	9 (0.5)	22 (1.2)	1848	210	3950
*Mucosal cancer* (*T1a*)	*415*	*0*	*2* (*0.5*)	*2* (*0.5*)	*1514*	*49*	*1978*
(B) Advanced cancer (T2–T4)	4344	37 (0.9)	37 (0.9)	105 (2.4)	2	2282	6628
(C) Not specified	11	1 (9.1)	0	1 (9.1)	1	0	12
2. Multiple primary cancers	1050	7 (0.7)	7 (0.7)	21 (2.0)	520	338	1908
1) Synchronous	587	4 (0.7)	2 (0.3)	10 (1.7)	210	185	982
(1) Head and neck	184	0	0	1 (0.5)	84	59	327
(2) Stomach	226	2 (0.9)	0	4 (1.8)	72	65	363
(3) Others	144	1 (0.7)	2 (1.4)	4 (2.8)	41	42	227
(4) Triple cancers	33	1 (3.0)	0	1 (3.0)	13	19	65
(5) Unknown	0	0	0	0	0	0	0
2) Metachronous	463	3 (0.6)	5 (1.1)	11 (2.4)	310	153	926
(1) Head and neck	102	0	1 (1.0)	2 (2.0)	107	38	247
(2) Stomach	114	2 (1.8)	1 (0.9)	3 (2.6)	75	36	225
(3) Others	221	1 (0.5)	2 (0.9)	5 (2.3)	86	60	367
(4) Triple cancers	26	0	1 (3.8)	1 (3.8)	42	19	87
(5) Unknown	0	0	0	0	0	0	0
Unknown	0	0	0	0	0	0	0

Values in parenthesis represent mortality %

*EMR* endoscopic mucosal resection (including endoscopic submucosal dissection)

Among benign esophageal diseases (Table 35), hiatal hernia, esophageal varices, esophagitis (including reflux esophagitis) and achalasia were the most common conditions in Japan. On the other hand, spontaneous rupture of the esophagus, benign esophageal tumors and congenital esophageal atresia were common diseases which were surgically treated as well as the above-mentioned diseases. The thoracoscopic and/or laparoscopic procedures have been widely adopted for benign esophageal diseases, in particular achalasia, hiatal hernia and benign tumors. Open surgery was performed in 1072 patients with a benign esophageal disease, with 30-day mortality in 14 (1.3 %), while thoracoscopic and/or laparoscopic surgery was performed for 588 patients, with 1(0.2 %) of the 30-day mortality The difference in these death rates between open and scopic surgery seems to be related the conditions requiring open surgery.

The majority of malignant diseases were carcinomas (Table 36). Among esophageal carcinomas, the incidence of squamous cell carcinoma was 90.5 %, while that of adenocarcinomas including Barrett cancer was 7.1 %. The resection rate for patients with a squamous cell carcinoma was 76.4 %, while that for patients with an adenocarcinoma was 88.3 %.

According to location, cancer in the thoracic esophagus was the most common (Table 37). Of the 3950 patients (37.3 % of total esophageal malignancies) having superficial esophageal cancers within mucosal and submucosal layers, 1892 (47.9 %) patients underwent esophagectomy, while 1848 (46.8 %) patients underwent EMR or ESD. The 30-day mortality rate and hospital mortality rate after esophagectomy for patients with a superficial cancer were 0.5 and 1.2 % respectively. Advanced esophageal cancer invading deeper than the submucosal layer was observed in 6628 (62.6 %) patients. Of the 6628 patients with advanced esophageal cancer, 4344 (65.5 %) underwent esophagectomy, with 0.9 % of the 30-day mortality rate, and with 2.4 % of the hospital mortality rate.

Multiple primary cancers were observed in 1908 (18.0 %) of all the 10,584 patients with esophageal cancer. Synchronous cancer was found in 982 (51.5 %) patients, while metachronous cancer (found before esophageal cancer) was observed in 926 (48.5 %) patients. The stomach is the commonest site for both synchronous and metachronous malignancy followed by head and neck cancer (Table 37).

**Fig. 4:**
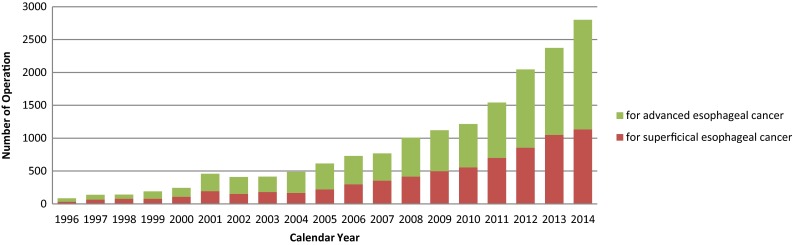
Annual trend of video-assisted esophagectomy for esophageal malignancy

Among esophagectomy procedures, transthoracic esophagectomy through right thoracotomy was the most commonly adopted for patients with a superficial cancer as well as for those with an advanced cancer (Table 38). Transhiatal esophagectomy commonly performed in Western countries was adopted in only 2.8 % of patients having a superficial cancer who underwent esophagectomy and in 1.6 % of those having an advanced cancer in Japan. The thoracoscopic and/or laparoscopic esophagectomy were adopted for 1134 patients (59.9 %) with a superficial cancer, and for 1666 patients (38.3 %) with an advanced cancer. The number of cases of thoracoscopic and/or laparoscopic surgery for superficial or advanced cancer has been increasing for these several years (Fig. 4).

**Table 53:** **Table 38** Malignant esophageal disease (surgical procedures)

	Operation (+)				Thoracoscopic and/or laparscopic procedure				EMR or ESD
	Cases	Hospital mortality			Cases	Hospital mortality			
		~30 days	31–90 days	Total (including after 91 days mortality)		~30 days	31–90 days	Total (including after 91 days mortality)	
Superficial cancer (T1)	*1892*	*9* (0.5)	*9* (0.5)	*22* (1.2)	*1134*	*3* (0.3)	*7* (0.6)	*14* (1.2)	1848
*Mucosal cancer* (*T1a*)	*415*	*0*	*2* (0.5)	*2* (0.5)	*223*	*0*	*0*	*0*	*1514*
Esophagectomy	*1892*	*9* (0.5)	*9* (0.5)	*22* (1.2)	*1134*	*3* (0.3)	*7* (0.6)	*14* (1.2)	1848
(1) Transhiatal esophagectomy	53	1 (1.9)	1 (1.9)	2 (3.8)	4	0	0	0	
(2) Transthoracic (rt.) esophagectomy and reconstruction	1579	5 (0.3)	8 (0.5)	17 (1.1)	1037	2 (0.2)	7 (0.7)	13 (1.3)	
(3) Transthoracic (lt.) esophagectomy and reconstruction	43	0	0	0	7	0	0	0	
(4) Cervical esophageal resection and reconstruction	35	0	0	0	16	0	0	0	
(5) Two-stage operation	27	0	0	0	13	0	0	0	
(6) Others	155	3 (1.9)	0	3 (1.9)	57	1 (1.8)	0	1 (1.8)	
(7) Not specified	0	0	0	0	0	0	0	0	
Advanced cancer (T2–T4)									
Esophagectomy	*4344*	*37* (0.9)	*37* (0.9)	*105* (2.4)	*1666*	*11* (0.7)	*11* (0.7)	*32* (1.9)	2
(1) Transhiatal esophagectomy	68	0	1 (1.5)	1 (1.5)	7	0	0	0	
(2) Transthoracic (rt.) esophagectomy and reconstruction	3661	31 (0.8)	26 (0.7)	78 (2.1)	1522	9 (0.6)	10 (0.7)	27 (1.8)	
(3) Transthoracic (lt.) esophagectomy and reconstruction	137	1 (0.7)	2 (1.5)	3 (2.2)	14	0	0	0	
(4) Cervical esophageal resection and reconstruction	171	1 (0.6)	2 (1.2)	8 (4.7)	35	1 (2.9)	0	2 (5.7)	
(5) Two-stage operation	84	1 (1.2)	1 (1.2)	4 (4.8)	25	0	0	0	
(6) Others/not specified	223	3 (1.3)	5 (2.2)	11 (4.9)	63	1 (1.6)	1 (1.6)	3 (4.8)	
(7) Not specified	0	0	0	0	0	0	0	0	
(Depth not specified)	*11*	*1* (9.1)	*0*	*1* (9.1)	*0*	*0*	*0*	*0*	1
Combined resection of other organs	*330*	*6* (1.8)	*4* (1.2)	*13* (3.9)					
(1) Aorta	2	0	0	0					
(2) Trachea, bronchus	24	0	0	1 (4.2)					
(3) Lung	77	3 (3.9)	2 (2.6)	6 (7.8)					
(4) Others	227	3 (1.3)	2 (0.9)	6 (2.6)					
Unknown	0	0	0	0					
Salvage surgery	*262*	*4* (1.5)	*4* (1.5)	*10* (3.8)	*55*	*0*	*2* (3.6)	*2* (3.6)	26

Values in parenthesis represent mortality %

Combined resection of the neighboring organs during resection of an esophageal cancer was performed in 330 patients (Tables 38, 39). Resection of the aorta together with the esophagectomy was performed in 2 cases. Tracheal and/or bronchial resection combined with esophagectomy was performed in 24 patients, with the 30-day mortality rate at 0 % and the hospital mortality rate at 4.2 %. Lung resection combined with esophagectomy was performed in 77 patients, with the 30-day mortality rate at 3.9 % and the hospital mortality rate at 7.8 %.

**Table 54:** **Table 39** Mortality after combined resection of the neighboring organs

Year	Esophagectomy			Combined resection											
				Aorta			Tracheobronchus			Lung			Others		
	a	b	c (%)	a	b	c (%)	a	b	c (%)	a	b	c (%)	a	b	c (%)
1996	4194	120	2.86	7	3	42.86	24	0	0.00	50	2	4.00	78	4	5.13
1997	4441	127	2.86	1	0	0.00	34	5	14.71	56	1	1.79	94	3	3.19
1998	4878	136	2.79	4	0	0.00	29	0	0.00	74	1	1.35	128	2	1.56
1999	5015	116	2.31	5	0	0.00	23	2	8.70	68	0	0.00	122	1	0.82
2000	5350	81	1.51	2	0	0.00	23	2	8.70	69	0	0.00	96	1	1.04
2001	5521	110	1.99	1	0	0.00	26	1	3.85	83	3	3.61	99	2	2.02
2002	4904	66	1.35	3	1	33.33	20	2	10.00	63	0	0.00	63	1	1.59
2003	4639	45	0.97	0	0	0.00	24	2	8.33	58	0	0.00	88	1	1.14
2004	4739	64	1.35	2	0	0.00	17	0	0.00	59	5	8.47	119	2	1.68
2005	5163	52	1.01	1	0	0.00	11	1	9.09	67	1	1.49	73	1	1.37
2006	5236	63	1.20	0	0	0.00	17	0	0.00	62	2	3.23	122	3	2.46
2007	4990	60	1.20	0	0	0.00	25	1	4.00	44	1	2.27	138	2	1.45
2008	5124	63	1.23	0	0	0.00	17	1	5.88	48	1	2.08	185	0	0.00
2009	5260	63	1.20	0	0	0.00	19	2	10.53	58	2	3.45	211	3	1.42
2010	5180	45	0.87	2	0	0.00	33	0	0.00	58	0	0.00	245	5	2.04
2011	5430	38	0.70	4	0	0.00	26	0	0.00	41	0	0.00	179	5	2.79
2012	6055	47	0.78	2	0	0.00	23	1	4.35	69	0	0.00	240	1	0.42
2013	5824	41	0.70	2	0	0.00	44	0	0.00	77	1	1.30	156	3	1.92
2014	6247	47	0.75	2	0	0.00	24	0	0.00	77	3	3.90	227	3	1.32
Total	98,190	1384	1.41	38	4	10.53	273	20	7.33	1181	23	1.95	2663	43	1.61

*a* The number of patients who underwent the operation, *b* number of patients died within 30 days after operation, *c* % ratio of b/a, i.e., direct operative mortality

Salvage surgery after definitive (chemo-) radiotherapy was performed in 262 patients, with the 30-day mortality rate at 1.5 % and with the hospital mortality rate at 3.8 % (Table 38).

Last, in spite of the efforts of the Committee to cover wider patient populations to this annual survey, the majority of the institutions which responded to the questionnaire were the departments of thoracic or esophageal surgery. It should be noted that larger number of patients with esophageal diseases should have been treated medically and endoscopically. We should continue our effort for complete survey through more active collaboration with the Japan Esophageal Society and other-related societies.
